# Whole Genome Resequencing Reveals Origins and Global Invasion Pathways of the Japanese Beetle *Popillia japonica*

**DOI:** 10.1111/mec.70008

**Published:** 2025-07-02

**Authors:** Rebecca Funari, Elahe Parvizi, Claudio Cucini, Sara Boschi, Elena Cardaioli, Daniel A. Potter, Shin‐ichiro Asano, Duarte Toubarro, Luca Jelmini, Francesco Paoli, Antonio Carapelli, Angela McGaughran, Francesco Frati, Francesco Nardi

**Affiliations:** ^1^ Department of Life Sciences University of Siena Siena Italy; ^2^ Te Aka Mātuatua/School of Science University of Waikato Hamilton New Zealand; ^3^ Department of Entomology University of Kentucky Lexington Kentucky USA; ^4^ Research Faculty of Agriculture Hokkaido University Sapporo Japan; ^5^ Faculty of Science and Technology University of the Azores Ponta Delgada Portugal; ^6^ Department of Finance and Economics Cantonal Plant Health Service Bellinzona Switzerland; ^7^ CREA Research Centre for Plant Protection and Certification Florence Italy; ^8^ National Biodiversity Future Center (NBFC) Palermo Italy

**Keywords:** insect pest, invasion genomics, Japanese beetle, phylogeography, whole genome resequencing

## Abstract

Invasive species are an increasing global threat given their ability to rapidly spread and adapt to novel environments. The adverse ecological and economic impacts of invasive species highlight the critical need to understand the mechanisms that underpin invasion processes and success. The Japanese beetle, *Popillia japonica* , is an invasive pest of remarkable interest, as it feeds on hundreds of economically valuable plant species. It has been expanding outside of its native range in Japan since the first decades of the 20th century, colonising large areas of North America and, more recently, Europe. Here, we compared whole‐genome resequencing data from individuals encompassing the entire species distribution to study the geographic differentiation of *P. japonica* populations and reconstruct expansion routes from Japan to the USA and Europe. We found six genomically distinguishable clusters, corresponding to the approximate colonisation areas at a continental scale. Our analysis supported an ancestral divergence between South and North/Central Japan, with the latter being the source of the initial invasion to the USA. Coalescent simulations supported independent bridgehead events from the USA to the Azores and Italy. We also investigated possible signals of selection to better understand the adaptive mechanisms that underlie the invasion success of *P. japonica* . However, the absence of strong selection signatures suggested that the beetle's adaptive ability might be embedded in pre‐existing genomic features. Our comprehensive genome‐wide dataset allowed a detailed inference of the invasion process and may be useful in determining the origin of *P. japonica* individuals in future invasion events.

## 1 Introduction

The relentless increase in human activities during the last few centuries has facilitated the global transport of organisms, resulting in a higher frequency of biological invasions (Hulme et al. 2009; Seebens et al. 2018). Species that establish outside their native range and become invasive can cause severe damage to ecosystems, biodiversity, human health and the global economy (Kolar and Lodge 2001; Simberloff et al. 2013). Invasion scenarios can sometimes be complex due to the occurrence of multiple introductions from various source areas (Kolar and Lodge 2001). For example, invasions can originate from successfully introduced populations, especially in highly connected hubs, which serve as a source for subsequent invasions through bridgehead events (Lombaert et al. 2010; Bertelsmeier and Keller 2018). Over the past decades, population genomic approaches, particularly using whole‐genome resequencing data, have been incorporated in the field of invasion science (North et al. 2021; Matheson and McGaughran 2022). Investigating global genomic diversity and population structure of invasive species can provide insights into the evolutionary mechanisms underlying invasion and, together with the inference of invasion pathways, can facilitate the development of effective strategies for invasive species prevention, management, and control (Estoup and Guillemaud 2010; North et al. 2021).

The Japanese beetle *Popillia japonica* Newman (Coleoptera: Scarabaeidae: Rutelinae) is an invasive insect native to Japan, where its presence is confirmed on all four major islands (Fleming 1972; EPPO 2022). The species was first reported outside its native range in Riverton, New Jersey (USA) in 1916; however, more recent investigations have traced the introduction back to 6 years earlier via a shipment of iris bulbs from Japan, whose roots were infested with grubs (Fleming 1972; Frank 2016). In 1944, *P. japonica* was detected in Canada near Halifax, Nova Scotia, possibly due to a range expansion from the USA (Althoff and Rice 2022; Strangi et al. 2023; Nardi et al. 2024). In 1970, the beetle was detected in Terceira Island (Azores) in the vicinity of the Lajes American military airbase; this was followed by further range expansion onto eight out of nine islands of the Azorean Archipelago over the following decades, including São Miguel and São Jorge, where *P. japonica* was first reported in 2003 and 2007, respectively (Simões 1984; EPPO 2019; Teixeira et al. 2024). The first record *of* *P. japonica* in continental Europe was in 2014 in Turbigo, Milano (Italy), in an area close to both the Milano‐Malpensa international airport and the Cameri military airport (Pavesi 2014). Rapidly expanding its distribution range, the Italian population crossed the border with Switzerland (Poggi et al. 2023; Strangi et al. 2023; Nardi et al. 2024), with the earliest report occurring in Stabio, Canton Ticino, in 2017 (Servizio fitosanitario cantonale 2017; Jelmini et al. 2022). The likely cause of the interceptions of a total of four specimens in North‐European countries (i.e., Germany and Netherlands) in 2018, 2021 and 2022 was occasional human‐mediated transport, while present records in other countries (e.g., China, Taiwan, South Korea and India) are considered invalid or misidentifications (EPPO 2022).

*Popillia japonica* is considered a major agricultural pest due to its highly polyphagous feeding behaviour on over 400 species of wild and cultivated plants (Althoff and Rice 2022; Poggi et al. 2022; Tayeh et al. 2023). While the larval diet is based on plant roots, adults thrive on foliage, flowers, and fruits, potentially affecting native biota (Potter and Held 2002; Baker and Potter 2018). The species is univoltine, with adults first emerging, depending on latitude and annual temperatures, between May and June (except for some colder areas where a generation takes 2 years to complete) and active through August (Fleming 1972; Potter and Held 2002). *P. japonica* is also able to expand rapidly from newly invaded areas, with an estimated range increase rate of approximately 10 km per year (Fleming 1972; Mondino et al. 2022; Poggi et al. 2023). A recent study on habitat suitability modelling suggests that the Eastern USA and the Azores archipelago are highly suitable areas for this species and that Central Europe, including the currently invaded areas, has a high risk of further invasion across the entire continent (Borner et al. 2023). Suitability estimates also predict the threat to worsen due to climate change, as increasing global temperatures might cause a shift from biannual to annual life cycles in cooler areas (Kistner‐Thomas 2019). Despite control strategies that have been effective, *P. japonica* continues to pose a significant threat to agriculture because of the diverse damage caused by larvae and adults and increasing restrictions in insecticide use, with only a few cases of successful local eradication (Potter and Held 2002; Althoff and Rice 2022; Gotta et al. 2023). In the USA alone, the economic impact of damage and control exceeds $460 million per year, while in Europe estimates of future damage to crops in the absence of management vary between €30 million and €7.8 billion annually (USDA‐APHIS 2015; Straubinger et al. 2022).

Previous studies have investigated the invasion pathways of *P. japonica* through the analysis of complete sequences of mitochondrial genomes, microsatellite loci and cytochrome oxidase subunit I (*cox1*) and cytochrome B (*cytb*) mitochondrial genes (Strangi et al. 2023; Nardi et al. 2024). However, these studies do not account for the full variability of *P. japonica* populations. Here, we use high‐resolution genome‐wide single nucleotide polymorphisms (SNPs) based on whole genome resequencing (WGR) data that can be particularly well‐suited to population genomic analyses involving recent introduction events (North et al. 2021). Our goal is to provide insight into the global genomic diversity and population structure of *P. japonica* , as well as infer the pathways of the more than 100‐year history of human‐mediated invasion of the species through demographic modelling. Finally, for the first time in this species, we investigate the presence of selection signatures by taking advantage of a recently annotated genome (Cucini et al. 2024) and performing genome‐scan analyses to identify allele frequency divergence between source and invasive populations to identify genes putatively under selection.

## 2 Materials and Methods

### 2.1 Sample Collection and Whole Genome Resequencing

Individuals of *P. japonica* were collected across its entire range to ensure a comprehensive representation and a balanced number of individuals across the four major areas in which the species is distributed (Japan, North America, the Azores, and Italy + Ticino). Within each major area, we collected samples from multiple locations to maximise geographic coverage.

DNA extraction, Illumina library preparation and sequencing followed the procedures as described in Nardi et al. (2024). Briefly, total DNA was extracted from dissected male testes of 81 individuals using Wizard Genomic DNA Purification kits (Promega) and sequenced at Macrogen Europe (The Netherlands) using TruSeq DNA PCR free libraries (Illumina) and a paired end (PE) 150 bp strategy, targeting 20 GB of sequence data per individual (*n* = 81 individuals). Three additional individuals from Cucini et al. (2024), and one individual from the Canseq150 program (SRR8479473, unpublished) were also included, with their raw data down sampled to 20 GB for consistency. Following the removal of two individuals with sub‐optimal sequencing data, a total of 83 individuals were successfully processed. These included the ancestral area of the species (i.e., Japan, including six locations in three islands, *n* = 21) and invasive populations in North America (11 locations in USA and Canada, *n* = 21), the Azores (two locations in two islands, *n* = 20), and Italy and Ticino (five locations, *n* = 21) (Table S1).

### 2.2 Variant Calling and Filtering

Quality control of raw sequence data was performed in FastQC v. 0.11.9 (Andrews 2010), followed by trimming in fastp v. 0.23.2 (Chen et al. 2018; head and tail trimming if *Q* < 20, sliding window trimming if *Q* < 24 over 4 bp). Trimmed sequences were mapped against the reference genome of *P. japonica* (6164 scaffolds, 578,347,224 bp total scaffold length; NCBI accession: JASPKY000000000; Cucini et al. 2024, available with metadata in FigShare under DOI: 10.6084/m9.figshare.27292584) using bbmap v. 35 (Bushnell 2014; maxindel = 200, pairlen = 500, other parameters at default). Duplicated reads were removed in picard v. 2.2.4 (http://broadinstitute.github.io/picard). Non‐matching pairs were identified and removed in samtools v. 1.11 (Danecek et al. 2021). Variant calling was performed in BCFtools v. 1.13 (Danecek et al. 2021; multiallelic caller, ploidy = 2, targeting both SNPs and indels). Only SNPs > 3 bp distant from an indel were retained.

Raw variants were subject to a series of filtering steps. At the level of contigs/genomic regions, SNPs were removed if they were in: regions identified as repeats or corresponding to repeat elements; contigs < 1 kb; and contigs with a median coverage > 3 SD from the mean calculated over all contigs. We then performed extensive data exploration at the level of SNPs (retained if biallelic, with a site quality > 50, a site mean depth across individuals of 15–52, missing data per site < 5%, and a distance > 5 bp from a second SNP) and individuals (including mean depth across sites, missing sites per individual, and heterozygosity—no outliers identified) using VCFtools v. 0.1.16 (Danecek et al. 2011). The final dataset, hereafter referred to as snps_3p, was obtained by applying a minor allele frequency of 0.015, implying that a variant is retained if present in at least three chromosomes or two individuals. A second dataset, snps_3p_unlinked, was obtained following linkage pruning of the snps_3p dataset in plink v. 1.90b6.21 (Purcell et al. 2007; window size 50 kb, window step 10 kb, r^2^ threshold 0.1) to obtain a subset of unlinked markers present in at least three chromosomes.

### 2.3 Genome‐Wide Diversity

The *population* module of STACKS v. 2.64 (Rochette et al. 2019) was used to calculate average observed and expected heterozygosity (*H*_o_ and *H*_e_), and average inbreeding coefficient per population (*F*_IS_; values ranging from −1 to 1; Wright 1949). VCFtools was used for sliding window analysis within 5 kb non‐overlapping genomic windows, to evaluate overall nucleotide diversity (*π*) per population and Tajima's D (Tajima 1989). The significance of population differences in *π* was assessed using the Kruskal–Wallis test, and the Wilcoxon rank‐sum test with Bonferroni correction was used as a post hoc test. In addition, pairwise nucleotide diversity among individuals from each area was calculated as the average of per site‐*π* using VCFtools, with differences among groups tested using the Wilcoxon test with Bonferroni correction for multiple comparisons.

Linkage disequilibrium (LD) decay patterns were evaluated using PopLDdecay v. 3.42 (Zhang et al. 2019), by calculating the *r* ^2^ between SNPs in different population subsets for six randomly selected individuals per population (South Japan, North/Central Japan, USA + Canada, São Jorge, São Miguel, Italy + Ticino; see Results), with a maximum distance between SNPs of 100 kb. All genomic diversity analyses were conducted using the snps_3p dataset.

### 2.4 Population Structure and Differentiation

To evaluate the distribution of genetic variation, we performed a PCA using plink on the snps_3p_unlinked dataset and plotted the first two principal components using the R package ggplot2 v. 3.3.5 (Wickham 2016) in R v. 4.3.1 (R Core Team 2023).

To infer individual admixture proportions and structure within and among populations, the sparse non‐negative matrix factorization (sNMF) algorithm implemented in the R package LEA v. 3.2.0 (Frichot et al. 2014; Frichot and François 2015) was applied on the snps_3p_unlinked dataset to analyse an assumed number of ancestral populations (*K*), ranging from 1 to 10, with 10 repetitions per tested *K* value. The cross‐entropy criterion was used to evaluate the optimal number of ancestral populations, with a smaller value suggesting a more optimal *K* value (Figure S1). The optimal number of clusters was identified at *K* = 5; however, a range of *K* values was explored to examine possible hierarchical population structure (Lawson et al. 2018).

A maximum likelihood phylogeny was inferred with 2000 bootstrap replicates using IQtree v. 2.0.3 (Minh et al. 2020) and the snps_3p_unlinked dataset. The ModelFinder option in IQtree was used to identify the best‐fit model of nucleotide substitution according to a Bayesian information criterion, which was identified to be K3Pu + F + R4.

Population pairwise fixation indices (*F*_ST_) (Weir and Cockerham 1984) were calculated for the snps_3p dataset, using the R package StAMPP v. 1.6.3 (Pembleton et al. 2013) with 100 bootstraps. Covariance structure among population allele frequencies, resulting from shared demographic histories among populations (Olazcuaga et al. 2020), was explored by estimating the scaled covariance matrix of the population allele frequencies (*Ω*) using the BayPass v. 2.3 core model (Gautier 2015) with the snps_3p_unlinked dataset.

### 2.5 Demographic Inference

To understand the colonisation history of *P. japonica* invasive populations, demographic modelling was performed based on the site frequency spectra (SFSs) using Fastsimcoal 2 (Excoffier et al. 2013, 2021). To reflect the temporal sequence of invasion records and to progressively evaluate alternative sources of colonisation, a step‐by‐step strategy was applied to sequentially test invasion scenarios identifying the source of São Miguel (Azores), São Jorge (Azores), and Italy + Ticino. This approach allowed for the targeted evaluation of specific branches of the invasion history while keeping the remaining topology fixed, thereby optimising the use of available genetic information for each colonisation event. To assess the repeatability of the inferred scenarios, a bootstrap approach was applied on five independent subsets of 30,000 randomly sampled SNPs. Accordingly, subsets of the snps_3p_unlinked dataset were extracted and converted to SFS using easySFS (https://github.com/isaacovercast/easySFS), projecting the number of samples down in each population to maximise the number of retained SNPs while minimising missing data.

Following the population structure patterns (see Results) and previous studies (Strangi et al. 2023; Nardi et al. 2024), we assumed that the invasive lineage originated from North/Central Japan, excluding the possibility of South Japan as a source of invasion. Consequently, a total of 19 invasion scenarios were tested across three sequential steps (Figure S2). The first step included three populations (North/Central Japan, USA + Canada, and São Miguel); then, building on the scenarios identified in previous steps, São Jorge was added in the second, and Italy + Ticino in the third.

Introduction times were modelled to 1–10 years before their first report (see Introduction), assuming one generation per year (Potter and Held 2002). All demographic scenarios were characterised by a bottleneck event at the time of invasion for invasive lineages. Bottleneck duration was set to 1–6 generations, and the number of individuals during bottlenecks was set to 10–500. An absence of gene flow was hypothesized for all scenarios. Each model was evaluated with 50 independent runs, 50 conditional maximisation algorithm cycles, and 500,000 simulations for likelihood maximisation. To determine the model with the highest support at each step, the best runs from each model were compared using the Akaike information criterion (AIC), taking into account the number of parameters included in each model.

Parameter estimates (i.e., effective population size—*N*_e_, bottleneck duration and size, and time of introduction before first detection)—along with their confidence intervals—were obtained using a non‐parametric bootstrap approach under the best‐supported model from the demographic analysis. One hundred bootstraps, each matching the original dataset in size, were generated by resampling with replacement from the snps_3p_unlinked dataset. Due to the absence of a species‐specific nuclear mutation rate for *P. japonica* or closely related species, we applied a mutation rate of 2.1 × 10^−9^ substitutions per site per generation, originally estimated for the non‐biting midge *Chironomus riparius* (Oppold and Pfenninger 2017) and also successfully employed by Pélissié et al. (2022) for the Colorado potato beetle. The rate lies within the generally accepted range for insects (2 × 10^−9^ to 7 × 10^−9^; Allio et al. 2017). Each bootstrap replicate was evaluated in Fastsimcoal 2 with 50 independent runs, 30 cycles of the conditional maximisation algorithm, and 50,000 simulations for likelihood maximisation. Confidence intervals of maximum likelihood estimates were calculated following Marchi et al. (2024) by computing the 2.5% and 97.5% quantiles of the bootstrap distribution for each parameter.

### 2.6 Genome‐Wide Scans for Detection of Outlier SNPs and SNPs Annotation

Using the snps_3p_unlinked dataset, two approaches were applied to study putative genomic signatures of selection: outlier detection with the R package PCAdapt v. 4.3.3 (Privé et al. 2020) and an *F*_ST_ outlier calculation approach. Based on demographic inference and patterns of population structure (see Results), and consistent with the previously hypothesized sequence of introductions (Strangi et al. 2023; Nardi et al. 2024), the following population pairs were contrasted: (1) North/Central Japan with USA + Canada, (2) USA + Canada with São Miguel (Azores), (3) São Miguel (Azores) with São Jorge (Azores), (4) USA + Canada with São Jorge (Azores), and (5) USA + Canada with Italy + Ticino.

After running PCAdapt using Mahalanobis distances and default parameters, optimal principal components (*K*) were selected through a scree plot test following Cattell's rule (Cattell 1966), resulting in *K* = 2 for all contrasts. We then explored multiple correction methods (including q‐value, Benjamini–Hochberg procedure and Bonferroni procedure), and finally applied the more stringent Bonferroni procedure (adj‐*p* < 0.001) to identify a final set of reliable candidate SNPs.

*F*_ST_ outlier loci were identified through sliding window analysis using VCFtools, within 5 kb non‐overlapping genomic windows, selecting the top 0.1% of weighted *F*_ST_ values. Manhattan plots of the genome‐wide weighted *F*_ST_ values and Bonferroni adjusted *p*‐values of PCAdapt were plotted using the R qqman package v. 0.1.9 (Turner 2018). To evaluate the possible effects of non‐equilibrium invasion dynamics in shaping the observed *F*_ST_ values, expected *F*_ST_ values at different contrasts were obtained by simulation under neutral evolution within the best‐fitting demographic model identified above. Sequences including 295,396 unlinked SNPs—that is, matching the snps_3p_unlinked_dataset—were simulated using Fastsimcoal 2. We then applied the same *F*_ST_ sliding window analysis to the simulated dataset to generate the expected distribution. Only those SNPs detected by both methods were considered as outliers, and these were plotted in a Venn diagram using the R ggvenn package v. 0.1.10 (Yan 2023).

SnpEff v. 5.1 (Cingolani, Platts, et al. 2012) was used to annotate outlier SNPs and investigate potential functional downstream effects on genes and proteins. A SnpEff database was manually built from the *P. japonica* GFF file and reference genome (Cucini et al. 2024), which provided functional annotations from Pfam (Mistry et al. 2021) and Interpro (Paysan‐Lafosse et al. 2023). SnpEff was then run following the software's documentation. For a better understanding of obtained results, SnpSift v. 5.1 (Cingolani, Patel, et al. 2012) was applied to filter annotated variants and find putatively relevant ones.

## 3 Results

From the 83 *P. japonica* individuals sequenced, we obtained 3,666,428 and 295,396 SNPs in the snps_3p (hard‐filtered with linked SNPs) and snps_3p_unlinked (hard‐filtered with unlinked SNPs) datasets, respectively (available in FigShare under DOI: 10.6084/m9.figshare.27292584).

### 3.1 Genomic Diversity

**FIGURE 1:**
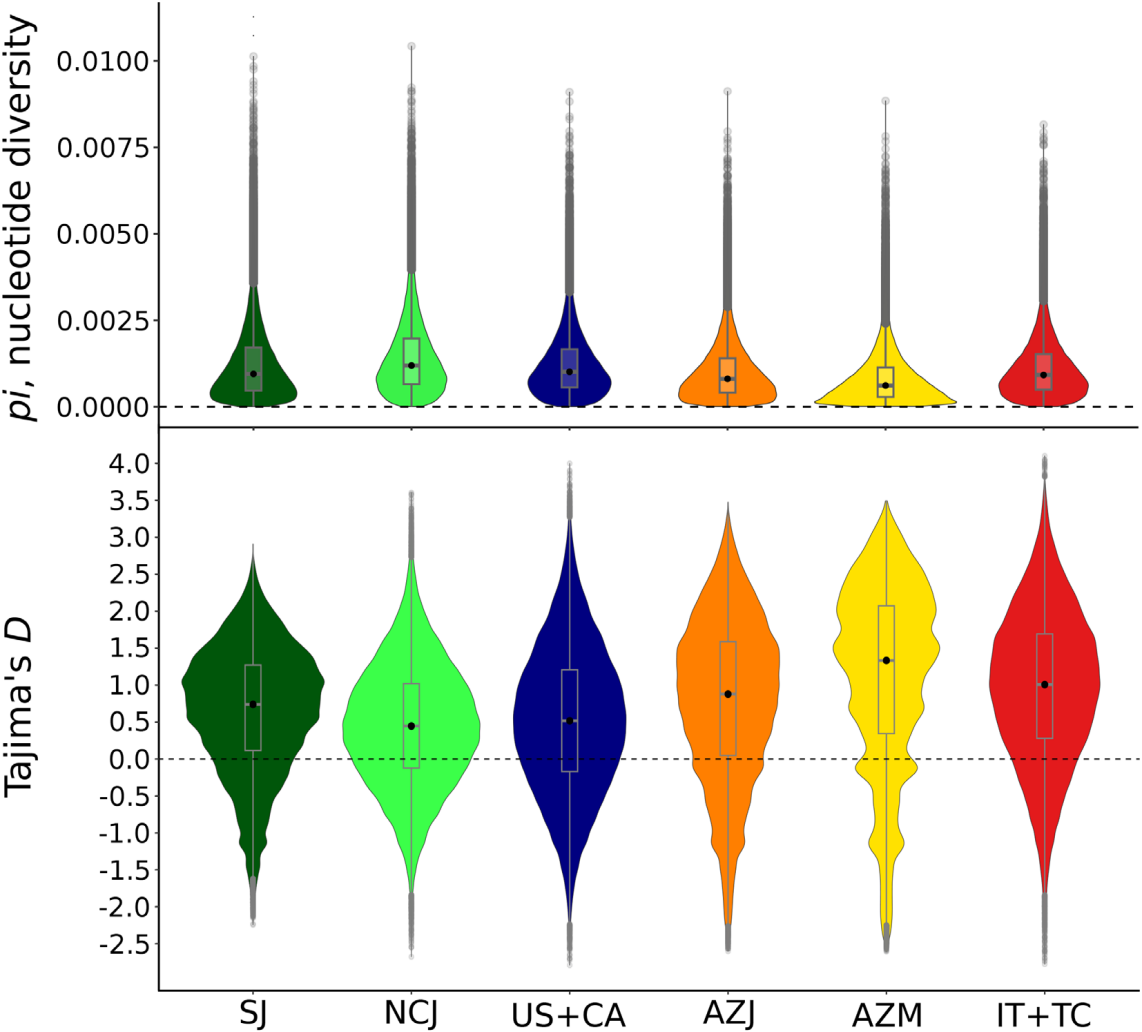
Per locus distribution of nucleotide diversity (top panel, *p*‐adj < 0.001 for each comparison), and Tajima's D (bottom), for each population of *Popillia japonica* , summarised across 5 kb genomic windows. AZJ, São Jorge (Azores); AZM, São Miguel (Azores); IT + TC, Italy + Ticino; NCJ, North/Central Japan; SJ, South Japan; US + CA, USA + Canada.

*Popillia japonica* populations showed low levels of nucleotide diversity (*π*) across native and invasive populations in sliding window analysis (Figure 1, top panel), with mean *π* ranging from 0.00083 [Standard Error (SE): 2.499e−06] for the Azorean population from São Miguel to 0.00144 (SE: 3.316e−06) for North/Central Japan. South Japan, USA + Canada, São Jorge and Italy + Ticino showed mean *π* of 0.00124 (SE: 3.239e−06), 0.00122 (SE: 2.781e−06), 0.00102 (SE: 2.607e−06), and 0.00112 (SE: 2.620e−06), respectively. On average, ancestral populations displayed higher diversity (mean *π* = 0.00134) than invasive ones (mean *π* = 0.00105) (Figure 1, top panel). The same pattern was confirmed for all populations through pairwise nucleotide diversity among individuals (Figure S3, *p*‐adj < 0.05 for all comparisons). Similarly, native populations showed higher observed heterozygosity (mean *H*_o_ = 0.1912 and 0.1548 in native and invasive populations, respectively) and a slight heterozygote deficit was observed in North/Central Japan and the USA (Table S2). The inbreeding coefficient was negative for the majority of populations, with the exception of North/Central Japan and the USA, which exhibited slightly positive estimates (Table S2).

Across populations, Tajima's D showed positive median genome‐wide estimates, ranging from 0.44630 to 1.33399 (Figure 1, bottom panel).

**FIGURE 2:**
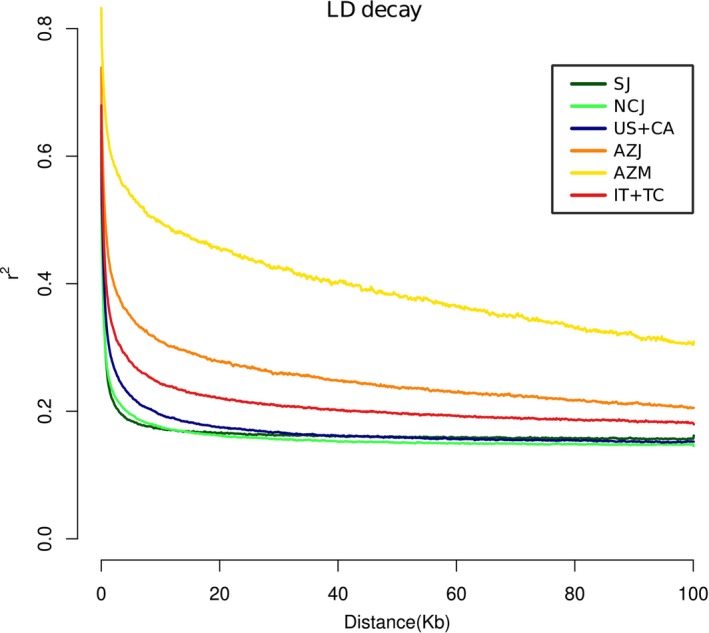
LD decay patterns in *Popillia japonica* populations, obtained by plotting LD decay estimates (*r* ^2^) within 100 kb genomic windows. AZJ, São Jorge (Azores); AZM, São Miguel (the Azores); IT + TC, Italy + Ticino; LD, linkage disequilibrium; NCJ, North/Central Japan; SJ, South Japan; US + CA, USA + Canada.

Linkage disequilibrium (LD) decay across 100 kb genomic windows was slowest for Azorean populations from São Jorge and São Miguel (Figure 2). Italy displayed an intermediate value, while LD decay was more rapid for both Japanese populations and for USA + Canada (Figure 2).

### 3.2 Population Structure and Differentiation

**FIGURE 3:**
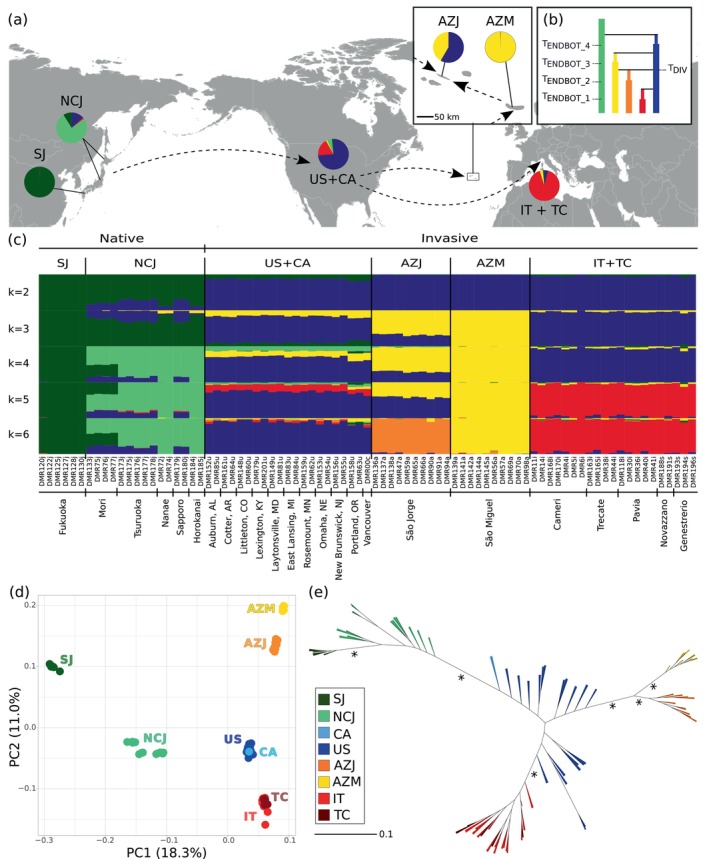
Global genomic structure of the Japanese beetle, *Popillia japonica* , based on 295,396 SNPs in 83 individuals. (a) Map of the distribution range and spread pathways of *P. japonica* ; pie charts of admixture proportions within all populations derived from the sNMF analysis at *K* = 5 [see also (c)]. (b) Best scenario from step‐by‐step demographic modelling analysis with Fastsimcoal 2. (c) Admixture plots of hierarchical population structure across different *K* values using the sNMF estimates. Each individual is represented by a thin vertical bar partitioned into *K*‐coloured segments representing estimated membership fractions of the individual in *K* clusters. (d) Principal component analysis showing variation in allele frequencies across populations. (e) Maximum likelihood unrooted tree showing phylogenetic relationships between native and invasive populations. Arrows indicate the pathways of invasion; asterisks mark key branches associated with major events, all with bootstrap values of 100. In (b), (d) and (e) populations are colour coded according to the key in (e). AZJ, São Jorge (Azores); AZM, São Miguel (Azores); CA, Canada; IT, Italy; IT + TC, Italy + Ticino; NCJ, North/Central Japan; SJ, South Japan; sNMF, sparse non‐negative matrix factorization; SNP, Single nucleotide polymorphism; TC, Ticino (Switzerland); *T*_DIV_, divergence time of admixed lineages; *T*_ENDBOT_, bottleneck end time; US, the USA; US + CA, USA + Canada.

The PCA identified six clearly differentiated genetic groups: South Japan, North/Central Japan, USA + Canada, São Jorge (Azores), São Miguel (Azores) and Italy + Ticino, with the first PC accounting for 18.3% of the total variation and showing differentiation between invasive and native clusters (Figure 3d). Investigating further population structure within Japan, North America and Europe, ancestral populations showed stronger spatial genetic differentiation than invasive populations. Some additional structure was observed within North/Central Japan, with locations Horokanai and Nanae (Hokkaido Island) clustering together, whereas Sapporo (Hokkaido) clustered with Tsuruoka (Honshu Island) (Figure S4). Mori (Honshu) was recovered as an independent cluster. No additional structure was visible within Italy + Ticino and USA + Canada (Figures 3d and S4).

Admixture analysis (sNMF) plots identified the same overall subdivision between ancestral (Japan) and invasive (all others) populations as the PCA. At *K* = 2, North/Central Japan and USA + Canada displayed some intermixing (Figure 3c). At *K* = 4–6, North/Central Japan shared some ancestry with South Japan and the USA. Overall, South Japan consistently stood out as well‐differentiated from the other populations, while North/Central Japan appeared to be the only population exhibiting internal structure, despite some weak signals for USA + Canada and Italy + Ticino at *K* = 10, in accordance with the results of other analyses in this study (Figures 3a and S5). Considering the invasive cluster, São Miguel emerged as the most genetically distinct population from *K* ≥ 3 (Figure 3a,c). São Jorge, in turn, exhibited some admixture between São Miguel and USA + Canada at *K* = 3–5 before becoming more differentiated at higher *K* values (Figures 3a,c and S5). In contrast, Italy + Ticino showed higher relatedness with USA + Canada (Figure 3a,c).

The phylogenetic tree of individuals clearly identified the same six genetic groups outlined above, with these all separated by fully supported nodes and South Japan diverging at the base of the tree (Figures 3e and S6). The branch leading to USA samples emerged from a paraphyletic group in North/Central Japan, with the two closest nodes representing individuals from Tsuruoka (Hokkaido Island). From within the USA clade, two clearly identifiable nodes branched off, one representing invasive individuals in the Azores (further subdivided among the two islands of São Jorge and São Miguel), and one representing invasive individuals in Italy + Ticino (Figures 3e and S6). The distribution of samples from the USA was subdivided in the tree, suggesting that the basal‐most individuals were localised in North‐Eastern USA, individuals at the base of the Azorean node were localised in Central/Eastern USA, while individuals rooting the Italy + Ticino node were mostly localised in Central/Western USA (Figure S6).

The highest genomic differentiation was observed between South Japan and invasive populations (*F*_ST_ ranging from 0.329 with the USA to 0.498 with São Miguel; Table S3). The USA displayed a lower genetic distance with both São Jorge and Italy + Ticino (*F*_ST_ = 0.102 and *F*_ST_ = 0.051, respectively), while between the latter a higher genomic distance was exhibited (*F*_ST_ = 0.152). Among invasive populations, São Miguel was the most differentiated (*F*_ST_ ranging from 0.185 with the other Azorean Island São Jorge to 0.252 with Italy + Ticino; Table S3).

A correlation matrix of population allele frequencies (*Ω*), summarising shared population history, confirmed the pairwise *F*_ST_ results (Table S4). For example, South Japan was poorly correlated with invasive populations (*Ω* ranging from 0.083 with USA + Canada to −0.022 with Italy + Ticino), and invasive populations revealed correlation patterns similar to those identified using *F*_ST_ estimates (Table S4). Indeed, USA + Canada exhibited high correlations with São Jorge and Italy + Ticino (*Ω* = 0.508 and *Ω* = 0.585, respectively), while São Jorge and Italy + Ticino were less correlated (*Ω* = 0.270). However, the matrix also clustered populations according to their origin, highlighting a strong division between native and invasive groups (Table S4).

### 3.3 Demographic History

Results of the demographic inference are reported in Figures 3b, S2, and S7; Table S5. In the first step (source of São Miguel), the best‐supported model—ModelA_1—identified the USA as the origin of São Miguel. Concerning the source of São Jorge, ModelB_5 received the strongest support and suggested its origin was derived through admixture between São Miguel—reflecting directional gene flow within the archipelago—and the USA. In the third step (source of Italy + Ticino), ModelC_1 was the best‐fitting, identifying the USA as the source of Italy + Ticino (Figures 3b and S2; Table S5). This overall pattern was consistent with analyses of population structure and the phylogenetic tree (see Section 3.2). Bootstrap analyses converged on the same scenario at all steps for all replicates, providing full support to our results. AIC values for the best‐supported models in the third and final step (4846.598, 5037.067, 5140.444, 5055.286, and 5183.917) were not always highly distant from those of the second‐best models (4849.256, 5038.524, 5142.214, 5055.950 and 5183.918, respectively) (Table S5). This limitation, not unexpected with very recent invasions, may have been further exacerbated by the use of a subset (~10%) of data in the bootstrap procedure. Variability of the second‐best model across bootstrap replicates, with models C_5, C_6 and C_7 alternating within a narrow AIC range, in contrast with the total consistency in the best scoring model across replicates, further supports our results (Figure S7 and Table S5).

Parameter estimates with confidence intervals associated with the best‐fitting model are provided in Table S6. Effective population sizes (*N*_e_) were lower in the invaded ranges than in the native North/Central Japan population. The estimated time from introduction to first detection, calculated as a single estimate across all invasive populations, indicated a lag of approximately 6 years, which was in line with the observations for the USA of Frank (2016). Bottleneck severity, assessed based on bottleneck duration and size, was relatively mild for USA + Canada, intermediate for Italy + Ticino, and strongest (i.e., smallest founding population sizes and longest bottleneck durations) for the Azorean populations.

### 3.4 Outlier SNPs and Annotated Genes

Comparison of observed *F*_ST_ values with *F*_ST_ values expected under neutral evolution given the demographic scenario described above produced values that are generally comparable, lending credibility to the threshold applied (Table S7). While all annotated outlier SNPs are reported below, only those arising from contrasts where observed *F*_ST_ was lower than the expected—that is, North/Central Japan with USA + Canada and São Miguel with São Jorge (Azores) – are discussed.

The PCAdapt and *F*_ST_‐based genome‐wide scans contrasting North/Central Japan with USA + Canada identified 710 and 201 outliers, respectively, with 17 outliers in common (Figure S8). Variant annotation showed that the majority of outlier SNPs in common with the two approaches were in intergenic regions (57.14%) and introns (28.57%), while the remaining SNPs were in upstream gene regions (9.52%), and downstream gene regions (4.76%).

The analysis contrasting USA + Canada with São Miguel identified 1367 and 182 outliers. Twenty‐eight were common to both methods (Figure S8), with variant annotation in intergenic (35.42%), intron (31.25%), upstream (16.67%), and downstream (14.58%) regions, and others in exons (as synonymous variants, 2.08%).

The contrast between São Miguel and São Jorge identified 138 and 139 outliers. Of these, 44 were common to both methods (Figure S8), with variant annotation in intergenic (39.02%), upstream (21.95%) and downstream (20.73%) regions, and exons (as synonymous variants, 2.44%).

Genome‐wide scans contrasting USA + Canada and São Jorge identified 1453 and 185 outliers, respectively, with four outliers in common between PCAdapt and *F*_ST_‐based methods (Figure S8). The variants were all in intergenic and upstream gene regions.

Finally, the contrast of USA + Canada and Italy + Ticino, did not identify shared outliers between methods (Figure S8). All outlier SNPs that received functional variant annotations from Pfam and Interpro for different domains and protein‐coding gene families, identified from the contrasts between invasive populations and their source populations, are listed in Table S8.

## 4 Discussion

In invasion biology, it is crucial to understand a species' history of invasion and geographic expansion routes. Here, we used genome‐wide SNP data from specimens that cover the entire species distribution to obtain high‐resolution genomic patterns for *P. japonica* and reconstruct invasion pathways at a continental scale. We identified distinguishable genomic clusters associated with independent invasion pathways. Population structure analysis, confirmed by demographic modelling, pinpointed the USA as the source of Azorean and continental European populations. Leveraging the new reference genome of *P. japonica* (Cucini et al. 2024), we also investigated possible signals of selection across various invasion routes of the species. We observed weak signals of selection in invasive populations, suggesting that invasion success in *P. japonica* has likely been underlain by standing genetic variation.

### 4.1 Spatial Genomic Patterns and Invasion Pathways

Population structure analyses clearly identified six genetic groups that correspond to separate geographic areas of the species distribution. These groups showed clear patterns of differentiation and diversity, with invasive populations (i.e., USA + Canada, São Jorge, São Miguel and Italy + Ticino) diverging from native Japanese populations (Figure 3). The native range, especially North/Central Japan, was characterised by higher *π* and *H*_*e*_ values than invasive populations, which matches the expectation of lower genetic diversity in the invasive range due to invasion‐associated demographic events such as founder effects and genetic bottlenecks (Michaelides et al. 2018; Comeault et al. 2020) (Figure S3 and Table S2). In invasive populations, positive Tajima's D and lower *π*, hinted at recent population size contractions and a loss of genetic diversity, as well as depletion of rare alleles during colonisation (Carlson et al. 2005; Comeault et al. 2020; Yang et al. 2022) (Figure 1). The slightly positive Tajima's D observed in the native range, in turn, might be the outcome of an ancient bottleneck, potentially associated with the last glaciation (see below; Nardi et al. 2024) (Figure 1, bottom panel).

São Miguel, São Jorge, and Italy + Ticino exhibited higher LD, indicating that these populations have not yet experienced extensive recombination, likely due to their recent introduction with small founding population sizes. In the invasive Azorean populations, and most noticeably in São Miguel, the detection of higher LD, lower nucleotide diversity, and elevated Tajima's D reflects a very small founder population at the time of introduction and consequent stronger single‐generation bottlenecks, in line with model parameter estimates related to bottleneck severity (Chen et al. 2021; Flanagan et al. 2021). In contrast, a more rapid LD decay, together with bottleneck‐related parameter estimates, pointed at a milder initial bottleneck in Italy +Ticino, alongside hints of ongoing population expansion, which had likely led to increased recombination (Slatkin 1994; Flanagan et al. 2021; Shan et al. 2023). Finally, the USA + Canada population, having experienced the mildest bottleneck according to our estimates of a larger founding population and shorter duration, and with over a century to recover and recombine, displayed an LD pattern more closely resembling native populations (Figure 2). The lower estimated effective population size in invasive populations compared to the demographically stable native range was consistent with founder effects and genetic drift, but might also be associated with recent bottlenecks in introduced populations (Charlesworth 2009).

Regarding the two native Japanese populations, our analyses confirmed previous research (mitochondrial genomes [Nardi et al. 2024], and microsatellites and two mitochondrial loci [Strangi et al. 2023]), showing ancestral divergence between the Southern population of Kyushu and Northern and Central groups. As suggested in Nardi et al. (2024), this separation probably occurred after the last glaciation when the previously connected Islands of Kyushu and Honshu (Central Japan) were divided by the formation of the Seto Sea, isolating the Southern population. This reconstruction also explains the strong genetic distance of this population with respect to all others that was observed in the present study. Indeed, population structure analyses confirmed North/Central Japan as the most likely source of the beetle's global invasion. Specifically, our results suggested Honshu Island as the original source, supporting the observations made by Strangi et al. (2023). However, given the structure observed within this population, it is worth noting that individuals from Tsuruoka in the Central Island that are genetically closer to the invasive group clustered with individuals from Sapporo in the Northern Island, obscuring the reliable identification of the invasion source at finer spatial scales.

A recent study of insect invasion histories underscore the critical impact of international trade networks in driving biological invasions (Sherpa and Després 2021). Numerous invasive insects originating from Eastern Asia, such as the mosquito *Aedes albopictus* , the fruit fly *Drosophila suzukii*, the harlequin ladybird *Harmonia axyridis* , and the brown marmorated stink bug *Halyomorpha halys* , have established themselves in North America and subsequently spread to Europe, reflecting a shared bridgehead invasion model (Lombaert et al. 2010; Parvizi et al. 2023; Sherpa and Després 2021). Our results unambiguously support that, subsequent to the invasion of the USA from Japan, the present distribution of non‐native populations of the Japanese beetle has been characterised by independent bridgehead events. Specifically, our findings indicate that the European invasions in the Azores and Italy originated from previously invaded areas of the USA, primarily supported by phylogenetic analysis and demographic inference. The latter also suggested that the USA is the source of São Miguel, which could have been facilitated by direct flights (data not shown). However, sNMF analysis revealed limited shared genomic ancestry between São Miguel and the USA, and pairwise *F*_ST_ and *Ω* estimations indicated high genetic distance between these populations. This may be explained by a small founding population in São Miguel and its current lower abundance compared to São Jorge (Teixeira et al. 2023), which might be leading to progressive genetic isolation. As for São Jorge, the demographic inference revealed that the population likely originated from admixture between the USA and São Miguel, which aligns with the sNMF and PCA analyses. However, the higher genetic distance between the two islands, observed in pairwise *F*_ST_ and *Ω* estimates relative to other source–invasive comparisons, suggests that São Miguel might not be the sole source of invasion in the Azores and it is possible that additional, unsampled populations have contributed to the invasion of São Jorge. In particular, greater connectivity and geographic proximity to other islands in the archipelago could have contributed to the genetic makeup of the São Jorge population.

While our genomic analyses agreed with those of previous studies, we found some discrepancies regarding the origin of European populations in the USA at finer spatial scales. For example, Nardi et al. (2024) observed a certain association between one mitochondrial genome in Maryland (East USA) and the Italian group, whereas Strangi et al. (2023), based on two mitochondrial loci, suggested the East coast of the USA and New Jersey as sources of invasion to the Azores and Italy, respectively. Our phylogenetic analysis based on SNPs from WGR suggested Central‐East USA as the most likely origin of the Azorean invasion, while little information could be obtained for the origin of the Italian outbreak, apart from a mild preference for Central‐West USA. We suggest that this lack of resolution may stem from extensive intermixing, recent invasions, and lack of structure within populations (also found in other studies; Nardi et al. 2024), preventing a confident reconstruction of invasion histories of European individuals at finer geographic scales, rather than from a limitation in the data. Lastly, our PCA, phylogeny, and admixture analyses supported the previously described hypothesis that the Japanese beetle has reached Canada and Ticino (Switzerland) from neighbouring countries (i.e., the USA and Italy) without post‐invasion genetic differentiation. This is plausible given the great hitchhiking and flight capacity of *P. japonica* (Fleming 1972; Allsopp 1996; Poggi et al. 2022; Borner et al. 2024).

### 4.2 Genomic Signatures of Selection and Adaptation

The majority of outlier SNPs that we identified in close proximity to genes could be associated with specific metabolism and cellular functions. However, to the best of our knowledge, their involvement in invasion and subsequent in situ adaptation of *P. japonica* and other insect pests remains unclear. Exceptions were one outlier SNP identified when contrasting North/Central Japan with USA + Canada, and five detected between the two Azorean populations, that were associated with genes encoding for ATP synthase, ABC transporters, galactosyltransferase, sulfatase, UDP‐glucosyl transferase, and UDP‐glucoronosyl transferase. The first seems to be involved in transmembrane ion transport, which has putative functions in insect acclimation and water homeostasis (Gáliková et al. 2018; Enriquez and Colinet 2019). ABC transporters, galactosyltransferase, and sulfatase have been previously associated with insecticide resistance and xenobiotic detoxification in invasive insects (Malka et al. 2016; Reid et al. 2019; Amezian et al. 2024). Similarly, UDP‐glucosyl transferase has been linked to insecticide resistance in the sweet potato whitefly *Bemisia tabaci* , while both UDP‐glucosyl transferase and UDP‐glucoronosyl transferase have been associated with cold acclimatisation in the maize caterpillar *Mythimna loreyi* (Yang et al. 2013; Duan et al. 2024).

It is worth noting that genome scans are subject to known limitations, as demographic effects can lead to potential false positives (Manel et al. 2016). However, the use of two complementary approaches combined with a stringent *p*‐value cut‐off threshold—both widely adopted in recent studies on WGR data for invasive species (e.g., Pélissié et al. 2022; Lu et al. 2024)—as well as the calculation of baseline *F*_ST_ accounting for the colonisation process, enhanced reliability of outlier detection.

Nevertheless, genome scan analysis identified isolated outliers and did not highlight typical peaks of differentiation in Manhattan plots. Consistent with Tajima's D results, this suggested an overall weak signal of in situ adaptation in invasive populations of *P. japonica* (Figures 1, S8, and S9). This pattern might be a consequence of the beetle's recent invasion history, with local adaptation perhaps more likely to occur long after establishment and spread (North et al. 2021) for this species. Additionally, it is well known that climatic and environmental conditions similar to those in the native distribution, extensive land use, and the highly‐generalist herbivore feeding habit of *P. japonica* have together favoured the spread of the beetle (Fleming 1972; Hamilton et al. 2007). Therefore, the absence of outlier SNPs between USA + Canada and Italy + Ticino may be attributed not only to the recent introduction of the European population from the USA, but also to a lack of novel ecological and environmental stressors, which may have facilitated the successful establishment and spread of *P. japonica* in the region. Borner et al. (2023) suggested that the majority of the present American and European infestation areas are moderately‐to‐highly suitable for the Japanese beetle. In addition, the recent investigation in Cucini et al. (2024) on *P. japonica* gene families involved in chemoreception and detoxification reported paralogous expansion in different subfamilies/clans of odorant receptors, ionotropic receptors, and cytochrome p450 encoding genes. This may suggest that some invasion‐facilitating characters, linked with the species' ability to feed on numerous plants and resist selective pressures from control and management measures, are already embedded in the Japanese beetle's genome. Adaptation from standing genetic variation might have contributed to this, potentially through rapid fixation of many alleles of small effect (Barrett and Schluter 2008). Such soft selective sweeps are increasingly known to underlie rapid evolutionary responses in invasive populations (Kołodziejczyk et al. 2025). While our stringent cut‐off for outlier detection was aimed at minimising false positive loci, we acknowledge that this approach may have reduced our ability to detect more subtle signals of selection arising from standing variation. Future studies using haplotype‐based genome scans or polygenic adaptation analyses may provide a more comprehensive picture of the mechanisms of local adaptation in *P. japonica* .

## 5 Conclusion

Genome‐wide SNP datasets and genomic tools in the current study allowed a detailed inference of the global invasion process of *P. japonica* . The identified geographic lineages, their genome‐wide variability and distribution worldwide, the relationships among populations, and the individual events of invasion of European territories from the USA were well resolved with our comprehensive dataset. Our research provides a robust genomic database against which genetic variation of future incursions can be compared to confidently pinpoint their origin at a global scale. Moreover, our dataset might help the design of surveillance strategies for integrated and targeted pest management practices in this species. Our results found an absence of strong selection taking place on invasive populations, suggesting that invasion success might be attributable to the presence of pre‐adaptive traits in *P. japonica* . However, to better understand the drivers of invasion success, future studies on *P. japonica* might focus on the identification and analysis of transposable elements and structural variants, since a growing body of literature has delved into their potential roles in fitness and adaptation during invasion (Carareto et al. 2014; Bertolotti et al. 2020; Mérot et al. 2020).

## Author Contributions

Antonio Carapelli, Francesco Frati and Francesco Nardi conceived the study. Sara Boschi, Elena Cardaioli and Rebecca Funari performed dissections and DNA extractions. Rebecca Funari, Francesco Nardi, Elahe Parvizi and Claudio Cucini analysed the data. Angela McGaughran supervised data analysis. Shin‐ichiro Asano, Luca Jelmini, Francesco Paoli, Daniel Potter and Duarte Toubarro provided the samples. Rebecca Funari, Francesco Nardi and Elahe Parvizi wrote the original draft. All authors reviewed and edited the manuscript.

## Disclosure

Benefit‐sharing statement. Benefits generated: benefits from this research accrue from the sharing of our data and results on public databases as described above.

## Conflicts of Interest

The authors declare no conflicts of interest.

## Supporting information


Data S1.

Table S1.

## Data Availability

Raw data were deposited in NCBI's SRA database within BioProject ID PRJNA860365, SRA numbers: SRR20647930, SRR20647937, SRR20647939, SRR2064794, SRR20647946 and SRR22354722‐SRR22354802, and Bio‐Sample numbers: SAMN29883562‐SAMN29883564 and SAMN31784955‐SAMN31785035. VCF files, the masked genome file alongside relevant metadata, as well as TPL and EST files are available on Figshare (DOI: 10.6084/m9.figshare.27292584). A description of the bioinformatic pipeline with code is available on GitHub (https://github.com/ESZlab/Population_genomics/tree/main/Popillia_japonica).
