# Supplementary material for: Whole Genome Resequencing Reveals Origins and Global Invasion Pathways of the Japanese Beetle Popillia japonica
Source: Mol Ecol. 2025 Jul 2;34(16):e70008. doi: 10.1111/mec.70008 (PMC12329639; doi:10.1111/mec.70008)

MOLECULAR ECOLOGY

Supplemental Information for:

Whole genome resequencing reveals origins and global invasion pathways of the Japanese beetle *Popillia japonica*

Rebecca Funari[1,2], Elahe Parvizi[2], Claudio Cucini[1], Sara Boschi[1], Elena Cardaioli[1], Daniel A. Potter[3], Shin-ichiro Asano[4], Duarte Toubarro[5], Luca Jelmini[6], Francesco Paoli[7], Antonio Carapelli[1,8], Angela McGaughran[2], Francesco Frati[1,8], Francesco Nardi*[1,8]

[1]*Dept. of Life Sciences, University of Siena, Italy;*

[2]*Te Aka Mātuatua/School of Science, University of Waikato, Hamilton, New Zealand;*

[3]*Dept. of Entomology, University of Kentucky, USA;*

[4]*Research Faculty of Agriculture, Hokkaido University, Japan;*

[5]*Faculty of Science and Technology, University of the Azores, Portugal;*

[6]*Dept. of Finance and Economics, Cantonal Plant Health Service, Switzerland;*

[7]*CREA Research Centre for Plant Protection and Certification, Florence, Italy;*

[8]*National Biodiversity Future Center (NBFC), Italy*

**francesco.nardi@unisi.it*

MOLECULAR ECOLOGY

Table S2. Estimates of population genomic indices in native and invasive populations of *Popillia japonica*, using 3,666,428 SNPs (snps_3p dataset) from 83 individuals. Native and invasive populations are coloured in green and yellow, respectively. H_o: average observed heterozygosity; H_e: average expected heterozygosity; F_{IS}: inbreeding coefficient; S.E.: standard error. Results obtained using STACKS software.

Populations	H_o	S.E.	H_e	S.E.	F_{IS}	S.E.
South Japan	0.1943	0.0001	0.1722	0.0001	-0.0112	0.0003
North/Central Japan	0.1881	0.0001	0.2078	0.0001	0.0757	0.0003
USA+Canada	0.1785	0.0001	0.179	0.0001	0.0181	0.0003
São Jorge (Azores)	0.1534	0.0001	0.1408	0.0001	-0.0116	0.0002
São Miguel (Azores)	0.1126	0.0001	0.1011	0.0001	-0.013	0.0002
Italy+Ticino	0.1746	0.0001	0.1665	0.0001	-0.0091	0.0004

Table S3. Pairwise population F_{ST} indicating genetic distance estimates between all populations of *Popillia japonica*, using 3,666,428 SNPs (snps_3p dataset) from 83 individuals. Native and invasive populations are coloured in green and yellow, respectively.

Populations	South Japan	North/ Central Japan	USA+ Canada	São Jorge (Azores)	São Miguel (Azores)
South Japan					
North/Central Japan	0.2374				
USA+Canada	0.3291	0.1352			
São Jorge (Azores)	0.4083	0.2081	0.1016		
São Miguel (Azores)	0.4976	0.2909	0.2057	0.1849	
Italy+Ticino	0.3657	0.1737	0.0506	0.1521	0.2524

MOLECULAR ECOLOGY

Table S4. Scaled covariance Ω matrix, estimating shared population history of *Popillia japonica,* using 295,396 SNPs (snps_3p_unlinked dataset) from 83 individuals. Native and invasive populations are coloured in green and yellow, respectively.

**Populations**	**South Japan**	**North/ Central Japan**	**USA+ Canada**	**São Jorge (Azores)**	**São Miguel (Azores)**	**Italy+ Ticino**
**South Japan**	1.0000					
**North/Central Japan**	0.6052	1.0000				
**USA+Canada**	0.0831	0.1238	1.0000			
**São Jorge (Azores)**	0.0190	0.0169	0.5078	1.0000		
**São Miguel (Azores)**	0.0469	0.0354	0.4106	0.7115	1.0000	
**Italy+Ticino**	-0.0223	-0.0426	0.5851	0.2700	0.2490	1.0000

MOLECULAR ECOLOGY

Table S5. Statistical estimates for models tested in the step-by-step demography using FastSimcoal2. A bootstrap approach was applied on five independent subsets of 30,000 randomly sampled SNPs. a) Source of São Miguel (Azores); b) Source of São Jorge (Azores); c) Source of Italy and Ticino. The best-fitting model for each bootstrap replicate, based on the lowest AIC, is highlighted in red. Abbreviations: MaxEstLhood, Maximum Estimated Likelihood; MaxObsLhood, Maximum Observed Likelihood; k, the number of estimated parameters; AIC, Akaike Information Criterion; Relative_Lhoods, relative likelihood.

a) Source of São Miguel (Azores)

Model	Subset	MaxEst Lhood	MaxObs Lhood	run	k	Best AIC	deltaAIC	Relative Lhoods	Akaike weights
modelA_1	Subset_I	-1177.457	-1145.377	16	9	5440.390	0	1	0.728
modelA_3	Subset_I	-1177.451	-1145.377	42	10	5442.362	1.972	0.373	0.272
modelA_2	Subset_I	-1190.668	-1145.377	34	9	5501.229	60.839	0.000	0.000
modelA_1	Subset_II	-1091.108	-1066.255	20	9	5042.738	0	1	0.531
modelA_3	Subset_II	-1090.728	-1066.255	27	10	5042.988	0.250	0.882	0.469
modelA_2	Subset_II	-1097.023	-1066.255	15	9	5069.978	27.240	0.000	0.000
modelA_1	Subset_III	-1065.327	-1039.215	1	9	4924.012	0	1	0.748
modelA_3	Subset_III	-1065.366	-1039.215	45	10	4926.192	2.180	0.336	0.252
modelA_2	Subset_III	-1073.025	-1039.215	28	9	4959.463	35.451	0.000	0.000
modelA_1	Subset_IV	-1183.352	-1151.47	21	9	5467.537	0	1	0.735
modelA_3	Subset_IV	-1183.361	-1151.47	40	10	5469.579	2.041	0.360	0.265
modelA_2	Subset_IV	-1193.726	-1151.47	46	9	5515.311	47.774	0.000	0.000
modelA_1	Subset_V	-1138.738	-1109.036	39	9	5262.082	0	1	0.699
modelA_3	Subset_V	-1138.67	-1109.036	48	10	5263.769	1.687	0.430	0.301
modelA_2	Subset_V	-1154.545	-1109.036	24	9	5334.876	72.794	0.000	0.000

b) Source of São Jorge (Azores)

Model	Subset	MaxEst Lhood	MaxObs Lhood	run	k	Best AIC	deltaAIC	Relative Lhoods	Akaike weights
modelB_5	Subset_I	-934.427	-914.562	47	15	4333.195	0	1	0.842
modelB_1	Subset_I	-936.028	-914.562	24	13	4336.568	3.373	0.185	0.156
modelB_6	Subset_I	-937.06	-914.562	26	15	4345.321	12.125	0.002	0.002
modelB_2	Subset_I	-946.264	-914.562	15	13	4383.707	50.511	0.000	0.000
modelB_4	Subset_I	-946.476	-914.562	38	15	4388.683	55.488	0.000	0.000
modelB_3	Subset_I	-959.627	-914.562	11	13	4445.246	112.050	0.000	0.000
modelB_5	Subset_II	-956.819	-933.631	3	15	4436.314	0	1	1.000
modelB_6	Subset_II	-964.34	-933.631	16	15	4470.950	34.635	0.000	0.000
modelB_2	Subset_II	-965.521	-933.631	26	13	4472.389	36.074	0.000	0.000
modelB_1	Subset_II	-966.536	-933.631	29	13	4477.063	40.748	0.000	0.000
modelB_4	Subset_II	-967.767	-933.631	27	15	4486.732	50.417	0.000	0.000
modelB_3	Subset_II	-985.287	-933.631	37	13	4563.414	127.100	0.000	0.000

modelB_5	Subset_III	-947.151	-922.62	13	15	4391.792	0	1	0.999
modelB_2	Subset_III	-951.088	-922.62	46	13	4405.922	14.131	0.001	0.001
modelB_4	Subset_III	-952.775	-922.62	17	15	4417.691	25.899	0.000	0.000
modelB_6	Subset_III	-953.907	-922.62	32	15	4422.904	31.113	0.000	0.000
modelB_1	Subset_III	-963.218	-922.62	35	13	4461.783	69.991	0.000	0.000
modelB_3	Subset_III	-964.503	-922.62	49	13	4467.700	75.909	0.000	0.000
modelB_5	Subset_IV	-1012.115	-982.291	18	15	4690.962	0	1	1.000
modelB_2	Subset_IV	-1017.479	-982.291	7	13	4711.664	20.702	0.000	0.000
modelB_4	Subset_IV	-1017.505	-982.291	32	15	4715.784	24.822	0.000	0.000
modelB_6	Subset_IV	-1020.276	-982.291	18	15	4728.545	37.583	0.000	0.000
modelB_1	Subset_IV	-1025.741	-982.291	50	13	4749.712	58.750	0.000	0.000
modelB_3	Subset_IV	-1031.183	-982.291	17	13	4774.773	83.811	0.000	0.000
modelB_5	Subset_V	-969.214	-944.41	49	15	4493.395	0	1	1.000
modelB_6	Subset_V	-973.939	-944.41	29	15	4515.155	21.759	0.000	0.000
modelB_2	Subset_V	-976.322	-944.41	21	13	4522.129	28.734	0.000	0.000
modelB_4	Subset_V	-976.529	-944.41	35	15	4527.082	33.687	0.000	0.000
modelB_1	Subset_V	-979.803	-944.41	28	13	4538.160	44.764	0.000	0.000
modelB_3	Subset_V	-989.269	-944.41	21	13	4581.752	88.357	0.000	0.000

c) Source of Italy and Ticino

Model	Subset	MaxEst Lhood	MaxObs Lhood	run	k	Best AIC	deltaAIC	Relative Lhoods	Akaike weights
modelC_1	Subset_I	-1089.924	-1058.519	34	18	5055.286	0	1	0.579
modelC_6	Subset_I	-1089.634	-1058.519	24	19	5055.950	0.665	0.717	0.415
modelC_7	Subset_I	-1091.698	-1058.519	14	19	5065.455	10.170	0.006	0.004
modelC_5	Subset_I	-1091.966	-1058.519	37	19	5066.689	11.404	0.003	0.002
modelC_2	Subset_I	-1098.288	-1058.519	41	18	5093.803	38.518	0.000	0.000
modelC_10	Subset_I	-1098.298	-1058.519	10	19	5095.849	40.564	0.000	0.000
modelC_9	Subset_I	-1098.35	-1058.519	42	19	5096.089	40.803	0.000	0.000
modelC_4	Subset_I	-1139.359	-1058.519	45	18	5282.942	227.657	0.000	0.000
modelC_8	Subset_I	-1141.14	-1058.519	26	19	5293.144	237.858	0.000	0.000
modelC_3	Subset_I	-1157.22	-1058.519	34	18	5365.195	309.910	0.000	0.000
modelC_1	Subset_II	-1117.856	-1080.278	31	18	5183.917	0	1	0.392
modelC_5	Subset_II	-1117.422	-1080.278	33	19	5183.918	0.001	0.999	0.392
modelC_7	Subset_II	-1117.679	-1080.278	32	19	5185.102	1.185	0.553	0.217
modelC_6	Subset_II	-1121.41	-1080.278	35	19	5202.284	18.367	0.000	0.000
modelC_2	Subset_II	-1131.914	-1080.278	41	18	5248.657	64.739	0.000	0.000
modelC_10	Subset_II	-1131.499	-1080.278	37	19	5248.745	64.828	0.000	0.000
modelC_9	Subset_II	-1131.553	-1080.278	34	19	5248.994	65.077	0.000	0.000
modelC_4	Subset_II	-1180.834	-1080.278	17	18	5473.942	290.024	0.000	0.000
modelC_8	Subset_II	-1181.309	-1080.278	42	19	5478.129	294.212	0.000	0.000
modelC_3	Subset_II	-1189.968	-1080.278	38	18	5516.005	332.088	0.000	0.000

MOLECULAR ECOLOGY

modelC_1	Subset_III	-1044.608	-1012.665	26	18	4846.598	0	1	0.721
modelC_6	Subset_III	-1044.751	-1012.665	11	19	4849.256	2.659	0.265	0.191
modelC_5	Subset_III	-1045.086	-1012.665	24	19	4850.799	4.201	0.122	0.088
modelC_7	Subset_III	-1047.296	-1012.665	26	19	4860.976	14.379	0.001	0.001
modelC_2	Subset_III	-1053.198	-1012.665	45	18	4886.156	39.558	0.000	0.000
modelC_10	Subset_III	-1053.269	-1012.665	45	19	4888.483	41.885	0.000	0.000
modelC_9	Subset_III	-1053.33	-1012.665	28	19	4888.764	42.166	0.000	0.000
modelC_4	Subset_III	-1096.542	-1012.665	41	18	5085.763	239.165	0.000	0.000
modelC_8	Subset_III	-1101.516	-1012.665	27	19	5110.669	264.071	0.000	0.000
modelC_3	Subset_III	-1117.975	-1012.665	30	18	5184.465	337.868	0.000	0.000
modelC_1	Subset_IV	-1085.968	-1049.925	31	18	5037.067	0	1	0.673
modelC_6	Subset_IV	-1085.85	-1049.925	41	19	5038.524	1.457	0.483	0.325
modelC_7	Subset_IV	-1088.241	-1049.925	32	19	5049.535	12.468	0.002	0.001
modelC_5	Subset_IV	-1088.375	-1049.925	10	19	5050.152	13.085	0.001	0.001
modelC_2	Subset_IV	-1095.784	-1049.925	28	18	5082.272	45.204	0.000	0.000
modelC_10	Subset_IV	-1095.711	-1049.925	7	19	5083.936	46.868	0.000	0.000
modelC_9	Subset_IV	-1095.769	-1049.925	8	19	5084.203	47.135	0.000	0.000
modelC_4	Subset_IV	-1125.616	-1049.925	27	18	5219.653	182.586	0.000	0.000
modelC_8	Subset_IV	-1136.584	-1049.925	3	19	5272.163	235.095	0.000	0.000
modelC_3	Subset_IV	-1153.174	-1049.925	39	18	5346.563	309.495	0.000	0.000
modelC_1	Subset_V	-1108.416	-1070.358	13	18	5140.444	0	1	0.500
modelC_7	Subset_V	-1108.366	-1070.358	40	19	5142.214	1.770	0.413	0.207
modelC_6	Subset_V	-1108.422	-1070.358	8	19	5142.472	2.028	0.363	0.182
modelC_5	Subset_V	-1108.634	-1070.358	23	19	5143.448	3.004	0.223	0.111
modelC_2	Subset_V	-1116.995	-1070.358	15	18	5179.952	39.508	0.000	0.000
modelC_9	Subset_V	-1116.621	-1070.358	43	19	5180.230	39.785	0.000	0.000
modelC_10	Subset_V	-1116.998	-1070.358	3	19	5181.966	41.522	0.000	0.000
modelC_8	Subset_V	-1160.169	-1070.358	28	19	5380.776	240.331	0.000	0.000
modelC_4	Subset_V	-1163.635	-1070.358	25	18	5394.737	254.293	0.000	0.000
modelC_3	Subset_V	-1173.807	-1070.358	20	18	5441.581	301.137	0.000	0.000

MOLECULAR ECOLOGY

Table S6. Confidence intervals for parameters estimated from 100 bootstrap replicates of the best-supported model identified in the step-by-step demographic analysis using FastSimcoal2. The 2.5th and 97.5th quantiles, around the maximum likelihood estimate, are shown. Estimated numbers of individuals were rounded to the nearest integer. Abbreviations: DETTIME, time from introduction to first detection; DURBOT, bottleneck duration; N_BOT, number of individuals during bottleneck; N_e, effective population size; Ncj, North/Central Japan; Usca, USA + Canada; Azm, São Miguel (Azores), Azj, São Jorge (Azores); Ittc, Italy + Ticino (Switzerland).

Estimated Parameter	Mean	2.5th quantile	97.5th quantile
DETTIME	5.51	2.48	8.00
DURBOT_Usca	3.55	1.48	5.00
DURBOT_Azm	5.20	4.00	6.00
DURBOT_Azj	3.78	2.00	6.00
DURBOT_Ittc	3.82	2.00	5.00
N_BOT_Usca	276	126	414
N_BOT_Azm	11	10	14
N_BOT_Azj	33	13	62
N_BOT_Ittc	58	27	91
N_e_Ncj	84376719	83783059	85051858
N_e_Usca	5290818	1403711	8560881
N_e_Azm	4993507	1722355	8427107
N_e_Azj	4764552	1411512	8520064
N_e_Ittc	5184316	1927680	8285342

MOLECULAR ECOLOGY

Table S7. F_{ST} values associated to individual invasion events contrasting source with invasive populations (mean and 99.99th quantile). The table shows observed values and values expected under neutral evolution and the best demographic scenario, as calculated in FastSimcoal2.

Contrasts between populations	Expected mean	Observed mean	Expected 999th quantile	Observed 999th quantile
North/Central Japan with USA + Canada	0.022	0.093	0.600	0.685
USA + Canada with São Miguel (Azores)	0.149	0.134	0.929	0.770
São Miguel (Azores) with São Jorge (Azores)	0.082	0.073	0.794	0.613
USA + Canada with São Jorge (Azores)	0.102	0.138	0.825	0.843
USA + Canada with Italy + Ticino	0.023	0.042	0.600	0.425

MOLECULAR ECOLOGY

Table S8. List of candidate genes (putatively under selection) derived from functional variant annotation analysis of 33 outlier SNPs, identified by F_{ST} and PCAdapt analyses and annotated as modifiers by SnpSift. Contrasts are as follows: North/Central Japan with USA+Canada (highlighted in blue), USA+Canada with São Miguel, Azores (highlighted in red), São Miguel, Azores with São Jorge, Azores (highlighted in green), and USA+Canada with São Jorge, Azores (highlighted in yellow). The analyses did not identify outliers in the contrast between USA+Canada and Italy+Ticino. SNP position indicates genomic scaffold and locus. Annotations (gene annotation, gene description, and gene function) were based on pfam and InterPro databases. InterPro accession numbers are indicated with one asterisk (*), and pfam accession numbers are indicated with two asterisks (**).

SNP position	Accession numbers	Gene Annotation	Gene description	Protein function
000044F:574340	IPR002404*, PF02174**	PTB domain (IRS-1 type)	Insulin receptor substrate (IRS)-type PTB domain	Protein binding
000159F:567053	IPR000194*, IPR004100*, PF00006**, PF02874**	ATP synthase alpha/beta family, nucleotide-binding domain ATP synthase alpha/beta family, beta-barrel domain	ATPase, F1/V1/A1 complex, alpha/beta subunit, nucleotide-binding domain	ATP binding, Transmembrane ion transport
	IPR003702*, IPR026888*, PF02550**, PF13336**	Acetyl-CoA hydrolase/transferase N-terminal domain Acetyl-CoA hydrolase/transferase C-terminal domain	Acetyl-CoA hydrolase/transferase	CoA-transferase activity
000207F:872553	IPR001611*, IPR000157*, PF13855**, PF13676**	Leucine rich repeat. TIR domain	Leucine rich repeat. Toll/interleukin-1 receptor homology (TIR) domain	Protein binding
000445F:99440	IPR007889*, PF05225**	helix-turn-helix, Psq domain	DNA binding HTH domain, Psq-type	DNA binding
000815F:137251	IPR004327*, PF03095**	Phosphotyrosyl phosphate activator (PTPA) protein	Phosphotyrosyl phosphatase activator	Phosphatase activator activity
000015F:304244	IPR002172*, PF01607**, PF00057**	Chitin binding Peritrophin-A domain. Low-density lipoprotein receptor domain class A	Low-density lipoprotein (LDL) receptor class A repeat	Protein binding
000034F:1659971	IPR000742*, IPR013032*, IPR011651*, IPR001774*, PF00008*,	EGF-like domain. Human growth factor-like EGF. N terminus of Notch ligand C2-like domain. Delta serrate ligand		Notch signaling pathway; multicellular organism development; cell

MOLECULAR ECOLOGY

	PF12661**, PF07657**, PF01414**			communication
000045F:432624	IPR000403*, IPR001263*, IPR002420*, PF00454**, PF00613**, PF00792**	Phosphatidylinositol 3- and 4-kinase. Phosphoinositide 3-kinase family, accessory domain (PIK domain). Phosphoinositide 3-kinase C2	Phosphatidylinositol 3-kinase catalytic subunit type 3	Signal transduction
	IPR015590*, PF00171**	Aldehyde dehydrogenase family	Aldehyde dehydrogenase, dimeric NADP-preferring	Oxidoreductase activity
000057F:326955	IPR013767*, IPR002073*, PF00989**, PF00233**	PAS fold. 3'5'-cyclic nucleotide phosphodiesterase		Regulation of DNA-templated transcription; signal transduction
000165F:203513	IPR031649*, IPR005821*, PF16905**, PF00520**	Voltage-dependent L-type calcium channel, IQ-associated. Ion transport protein	Voltage dependent calcium ion channel	Transmembrane ion transport
000168F:835680	IPR013162*, PF13927*, PF08205**	Immunoglobulin domain. CD80-like C2-set immunoglobulin domain	CD80-like, immunoglobulin C2-set	Immune response
000219F:117334	IPR004217*, PF02953**	Tim10/DDP family zinc finger	Zinc binding domain	Mitochondrial protein import
000232F:471102	IPR011701*, PF07690**	Major Facilitator Superfamily	Membrane transport protein	Transmembrane transport
000248F:49527	IPR002110*, IPR032425*, PF13857**, PF16511**	Ankyrin repeats. N-terminal or F0 domain of Talin-head FERM	Ankyrin repeat-containing domain	Protein binding
000292F:68431	IPR002123*, PF01553**	Acyltransferase	Phospholipid/glycerol acyltransferase	Acyltransferase activity
000293F:518778	IPR001356*, PF00046**	Homeodomain		DNA binding
00326F:192980	IPR004254*, PF03006**	Haemolysin-III related	AdipoR/Haemolysin-III-related	unspecified
000603F:266322	IPR002557*, IPR002172*, PF01607**, PF00057**	Chitin binding Peritrophin-A domain. Low-density lipoprotein receptor domain class A		Protein binding
000046F:449188	IPR002213* PF00201**	UDP-glucoronosyl and UDP-glucosyl transferase		UDP-glycosyltransferase activity
000088F:474595	IPR013120*, IPR033640*, PF07993**, PF03015**	Male sterility protein	Fatty acyl-coenzyme A reductase-like, NAD-binding domain	NAD binding

MOLECULAR ECOLOGY

	IPR041577*, PF17919**	RNase H-like domain found in reverse transcriptase	Reverse transcriptase/retrotransposon-derived protein, RNase H-like domain	RNA cleavage
000088F:659454	IPR042855*, PF00957**	Synaptobrevin	v-SNARE, coiled-coil homology domain.	Cell secretion
	IPR003439*, PF00005**	ABC transporter	ABC transporter-like, ATP-binding domain	ATP binding, ATP hydrolysis activity
	IPR011527*, PF00664**	ABC transporter transmembrane region	ABC transporter type 1, transmembrane domain	Transmembrane transport, ATP binding, ABC.type transporter activity
	IPR032410*, IPR001807*, PF16185**, PF00654**	Mitochondrial ABC-transporter N-terminal five TM region. Voltage gated chloride channel		
000124F:927672	IPR002659*, PF01762**	Galactosyltransferase	Glycosyl transferase, family 31	Protein glycosylation, hexosyltransferase activity
000137F:631514	IPR000917*, PF00884**	Sulfatase	Sulfatase, N-terminal	Sulfate hydrolysis
000137F:713153	IPR013842*, IPR000640*, PF06421**, PF00679**	GTP-binding protein LepA C-terminus. Elongation factor G C-terminus	GTP-binding protein LepA, C-terminal. Elongation factor EFG, domain V-like	GTP binding
	IPR004161*, IPR000795*, PF03144**, PF00009**	Elongation factor Tu domain 2. Elongation factor Tu GTP binding domain	Translation elongation factor EFTu-like, domain 2. Translational (tr)-type GTP-binding domain	GTP binding
	IPR044822*, PF13837**	Myb/SANT-like DNA-binding domain	Myb/SANT-like DNA-binding domain 4	DNA binding
	IPR001611*, PF13855**	Leucine rich repeat		Protein binding
000171F:232698	IPR007875*, PF05210**	Sprouty protein (Spry)		Multicellular organism development; Regulation of signal transduction
000245F:607784	IPR046360*, PF00907**	T-box	T-box transcription factor, DNA-binding domain	Positive regulation of DNA-templated transcription, DNA-binding transcription factor activity
000282F:23200	IPR002557*, PF01607**	Chitin binding Peritrophin-A domain		Protein binding

MOLECULAR ECOLOGY

000336F:450805	IPR006941*, PF04857**	CAF1 family ribonuclease	CCR4-associated factor 1 ribonuclease	mRNA deadenylation
000416F:492811	IPR000270*, IPR001478*, PF00564**, PF00595**	PB1 domain. PDZ domain	Phox and Bem1 domain. Discs-large homologous regions domain	Protein binding
000416F:500609	IPR000462*, PF01066**	CDP-alcohol phosphatidyltransferase		Phospholipid biosynthesis
000583F:317992	IPR001245*, PF07714**	Protein tyrosine and serine/threonine kinase	serine/threonine- and tyrosine-protein kinases, catalic domain	Protein phosphorylation; protein kinase activity
001052F:26312	IPR029526*, PF13843**	Transposase IS4	PiggyBac transposable element-derived proteins	Unspecified
000051F:1255341	IPR005135*, PF14529**	Endonuclease-reverse transcriptase	Endonuclease/exonuclease/phosphatase	Catalytic activity

MOLECULAR ECOLOGY

Figure S1. Cross-entropy results of sNMF runs for *Popillia japonica*. The run with the lowest cross-entropy value (K=5) represents the optimal population clustering scenario.

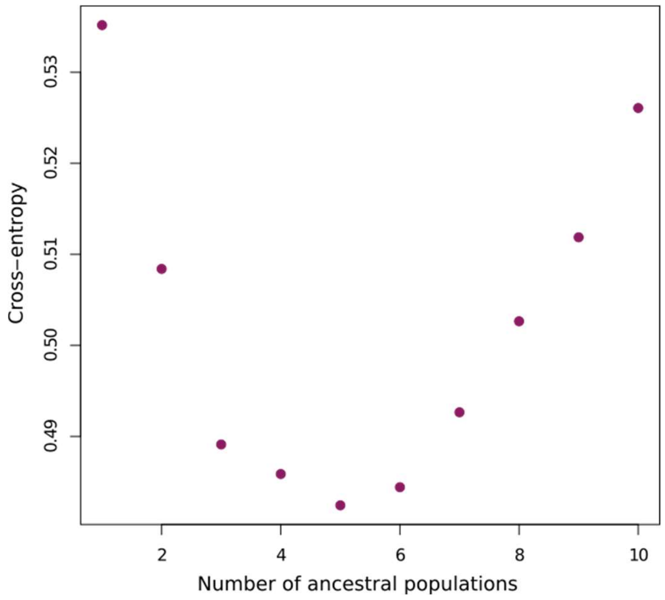

MOLECULAR ECOLOGY

Figure S2. Fastsimcoal2 models testing step-by-step invasion pathways of *Popillia japonica* in invaded areas, assuming North/Central Japan as the origin of the invasive lineage. All demographic scenarios were characterized by a bottleneck event at the time of invasion for invasive lineages and an absence of gene flow. Models highlighted in the red boxes were the most likely at each step, according to the AIC criterion. Abbreviations: T_{ENDBOT}, bottleneck end time; T_{DIV}, divergence time of admixed lineages; Azm, São Miguel (Azores); Azj, São Miguel (Azores); It+tc, Italy and Ticino.

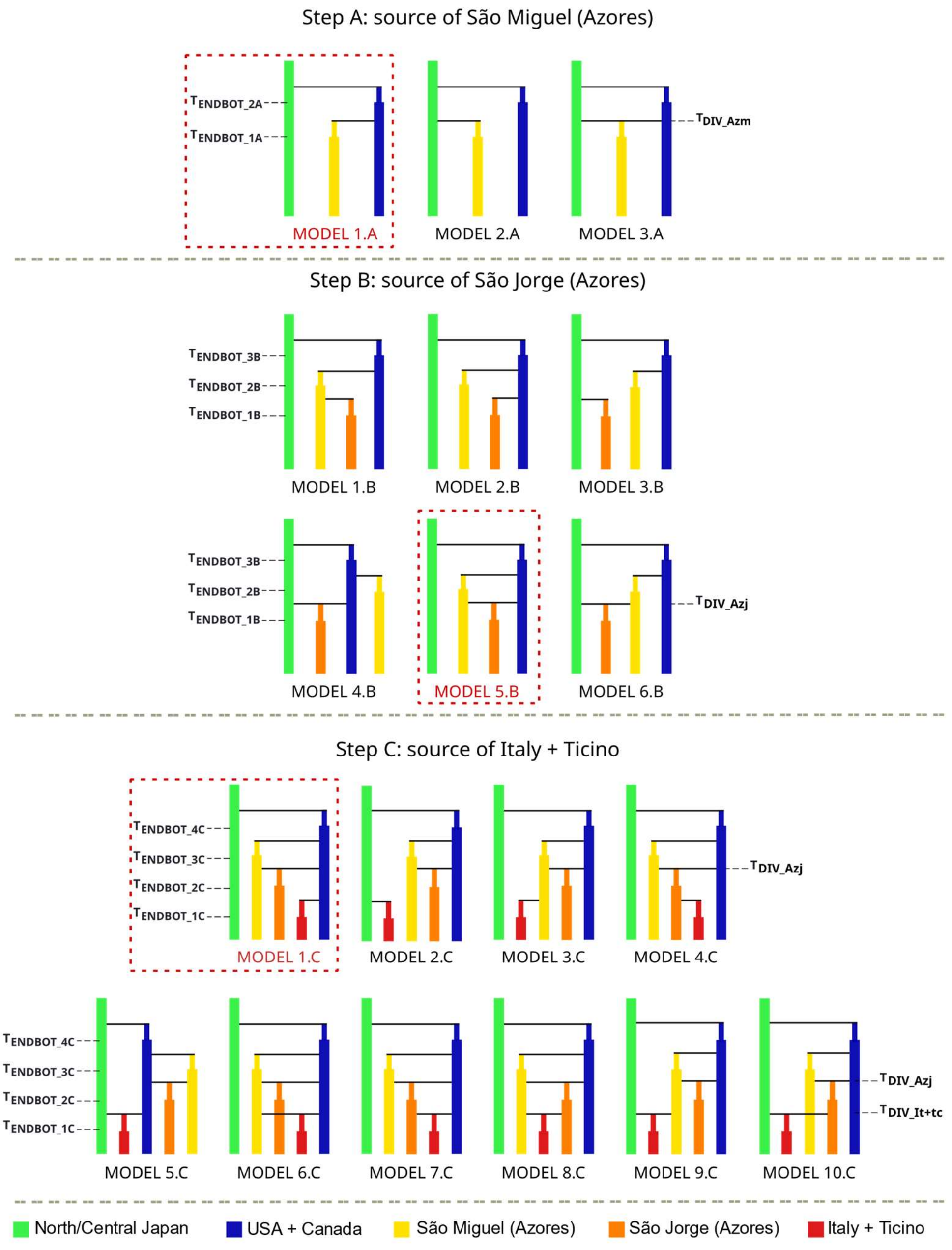

MOLECULAR ECOLOGY

Figure S3. Density plot of pairwise nucleotide diversity among individuals (p-adj < 0.05 for all comparisons), showing the distribution of average nucleotide diversity of values within each *Popillia japonica* population. Populations are color-coded according to the provided key.

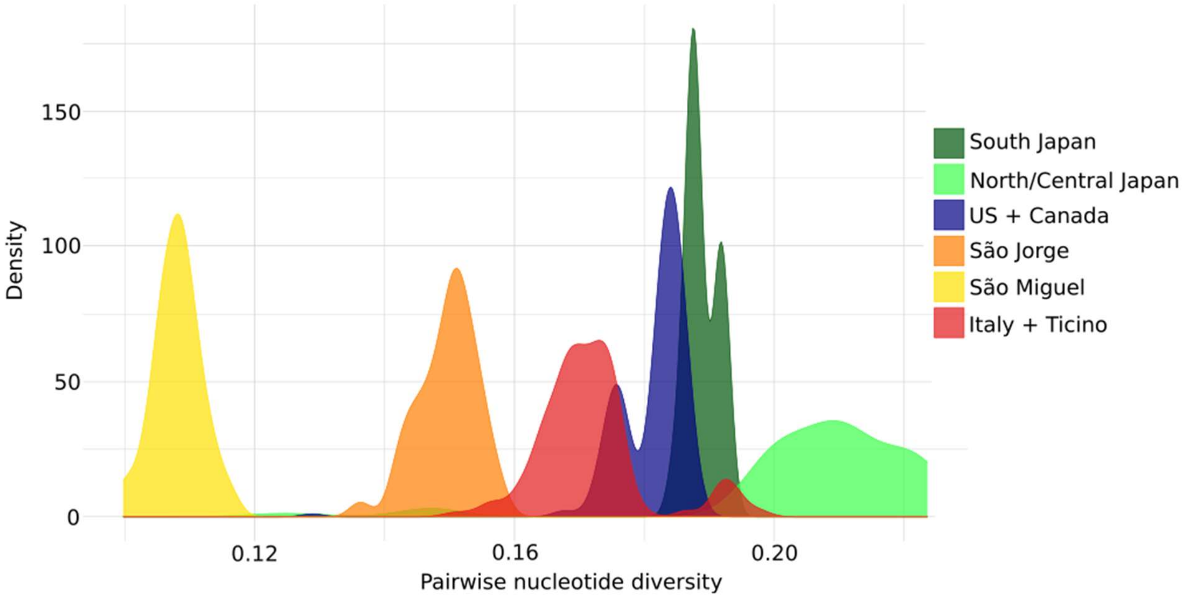

MOLECULAR ECOLOGY

Figure S4. Principal component analysis (PCA) insets for North/Central Japan, Italy+Ticino and USA+Canada, obtained by zooming in on the main PCA (Figure 3d) to show additional structure within North/Central Japan but not the other two main groups (Italy+Ticino, USA+Canada). *Popillia japonica* individuals are color-coded according to the keys to the right of each figure.

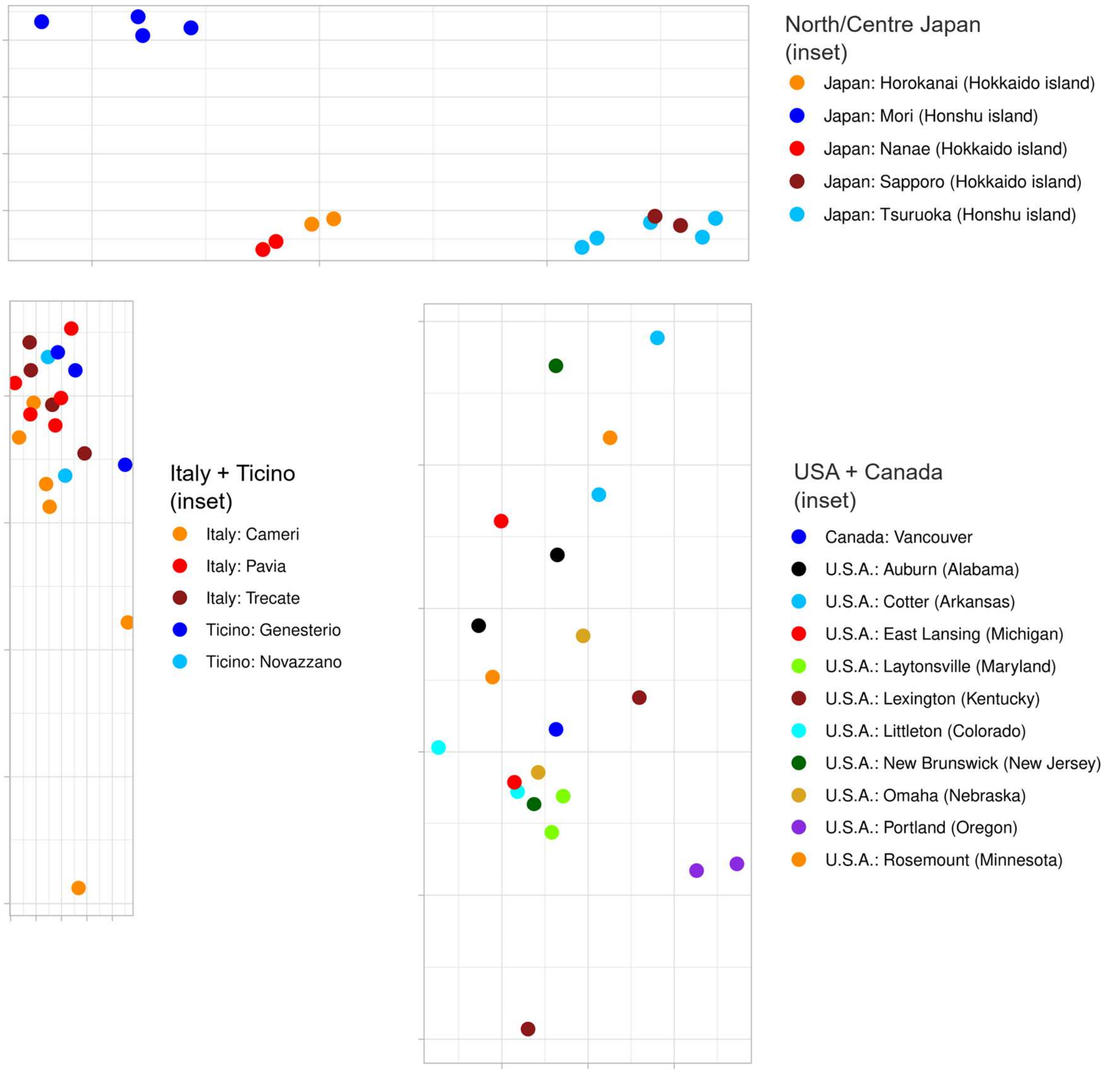

MOLECULAR ECOLOGY

Figure S5. Admixture plots derived from sNMF analysis confirming hierarchical population structure based on 295,396 SNPs from 83 individuals. Plots represent estimated ancestry coefficients of *Popillia japonica* populations across K=7-10.

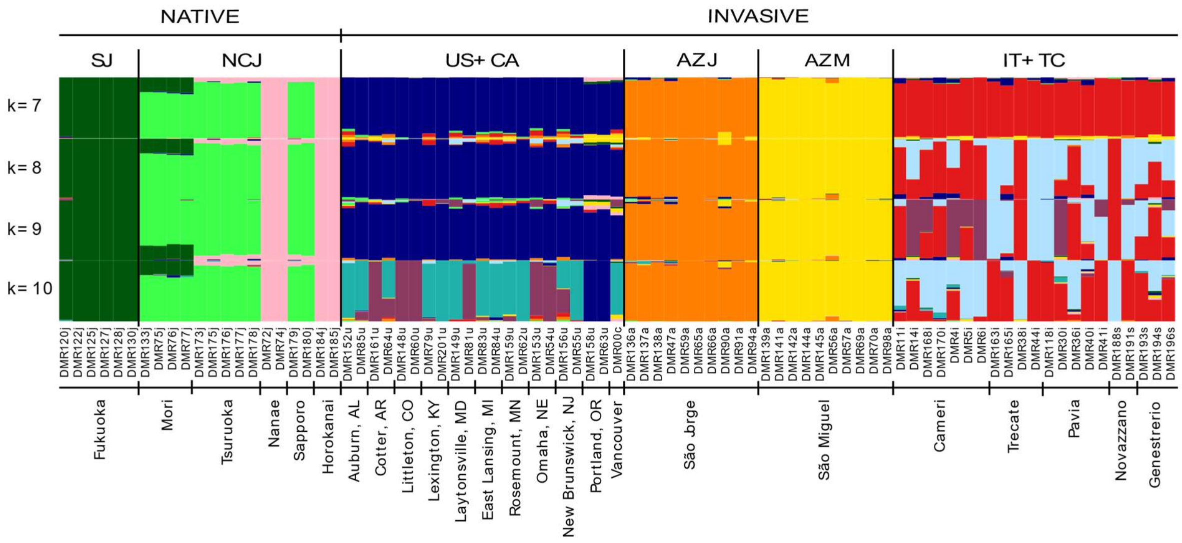

MOLECULAR ECOLOGY

Figure S6. Maximum likelihood rooted tree showing phylogenetic relationships between individual samples from native and invasive populations of *Popillia japonica*. The relationships between clades are depicted relative to South Japan (Kyushu). The tree was constructed using 295,396 SNPs from 83 individuals. The bootstrap support values for the key branches are highlighted in red. Populations are color-coded according to the provided key.

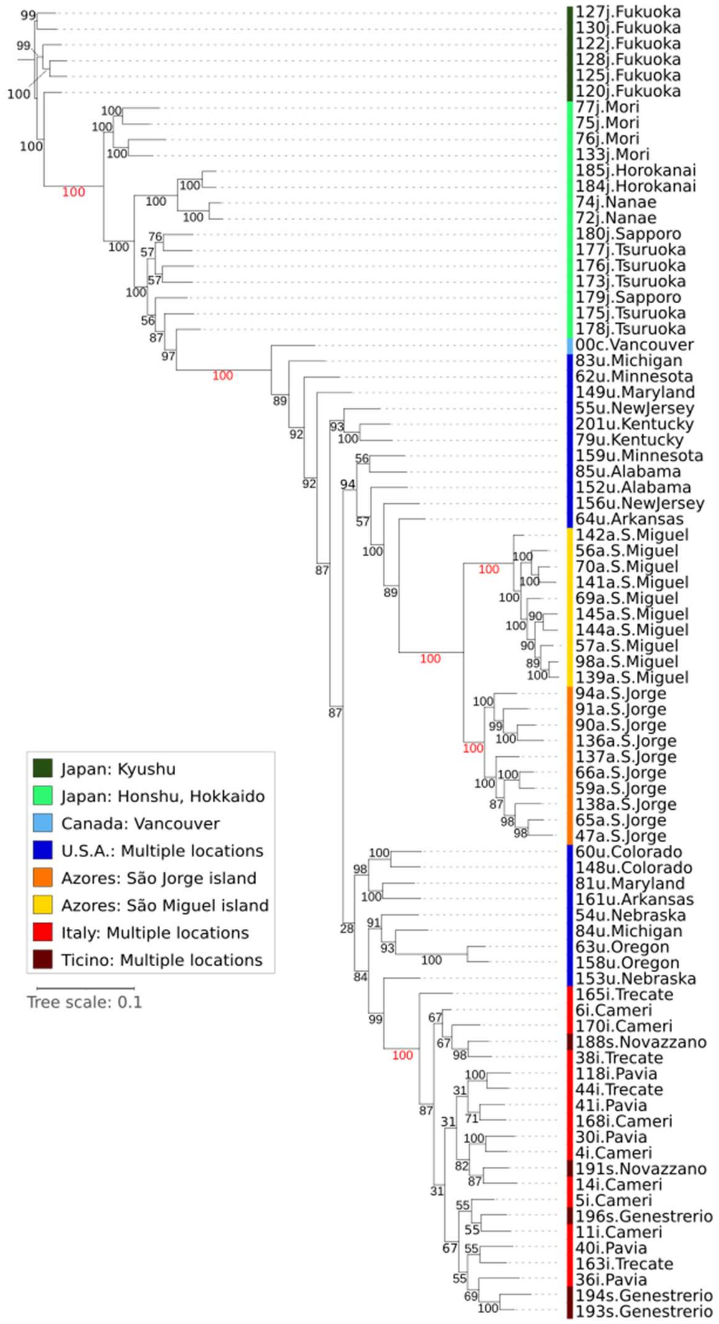

MOLECULAR ECOLOGY

Figure S7. Distribution of Akaike Information Criterion (AIC) values for each model and subset tested in the step-by-step demographic analysis using FastSimcoal2. a) Source of São Miguel (Azores); b) Source of São Jorge (Azores); c) Source of Italy and Ticino. Red dots indicate the run with the lowest (i.e., best) AIC value. A double asterisk (**) denotes the best-fitting model based on AIC. A blue x indicates the mean.

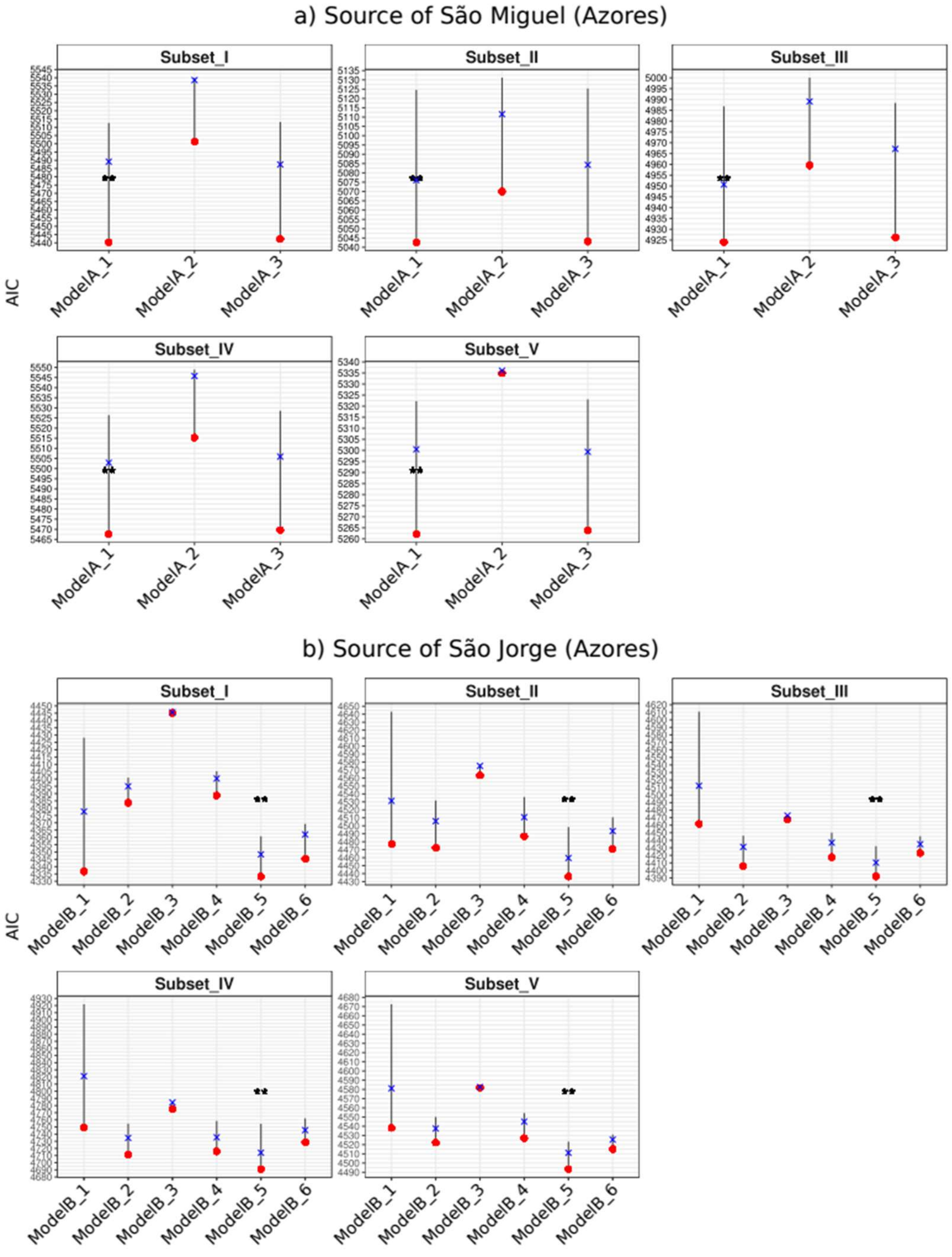

MOLECULAR ECOLOGY

c) Source of Italy and Ticino

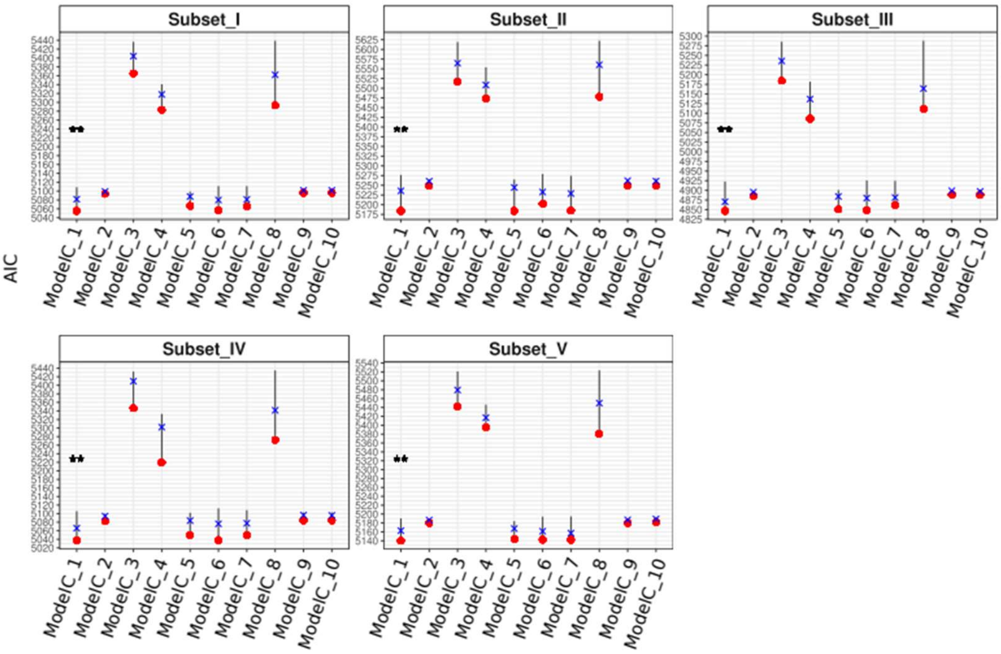

MOLECULAR ECOLOGY

Figure S8. Genome-scan analysis contrasting a) North/Central Japan with USA + Canada, b) USA + Canada with São Miguel (Azores), c) USA + Canada with São Jorge (Azores), d) São Miguel (Azores) with São Jorge (Azores), e) USA + Canada with Italy + Ticino, based on F_{ST} and PCAdapt. Manhattan plots display genome-wide weighted F_{ST} values, identified through sliding windows analysis within 5 kb non-overlapping genomic windows. The top 0.1% of SNPs are those above the red horizontal line, indicating putative loci under selection. The Venn diagram alongside each Manhattan plot shows the number of outlier SNPs between the two genome-scan analysis methods. The outlier SNPs detected by both F_{ST} and PCAdapt that received functional annotation are highlighted in red, while those without functional annotation are highlighted in green.

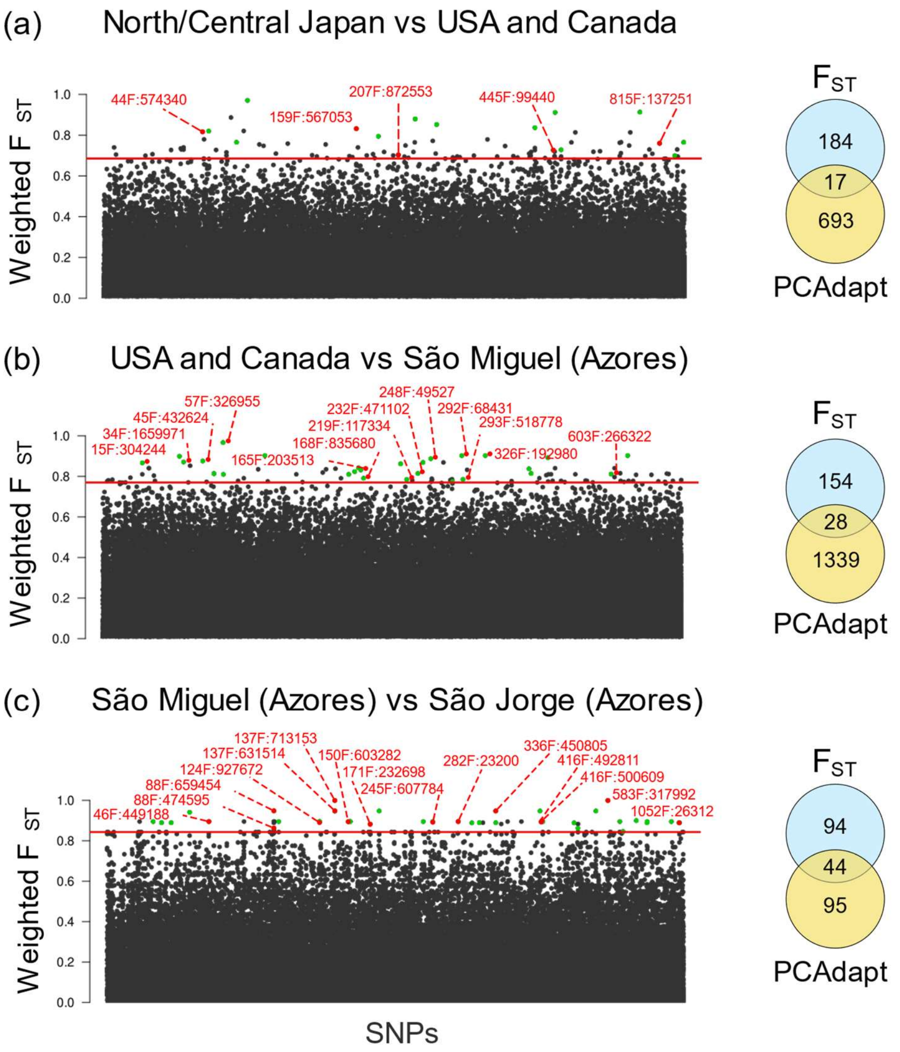

MOLECULAR ECOLOGY

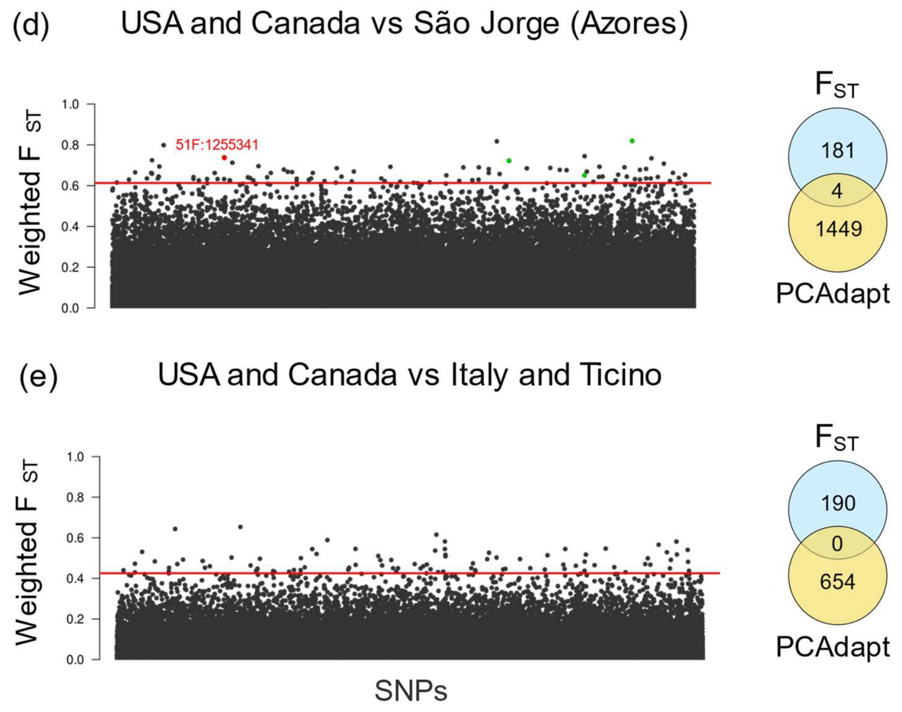

MOLECULAR ECOLOGY

Figure S9. Genome-scan analysis contrasting North/Central Japan with USA + Canada, USA + Canada with São Miguel (Azores), São Miguel (Azores) with São Jorge (Azores), USA + Canada with São Jorge (Azores), and USA + Canada with Italy + Ticino, based on F_{ST} and PCAdapt. Manhattan plots display the Bonferroni-adjusted significance threshold of P-value < 0.001 from PCAdapt. The top 0.1% of SNPs, indicated by those above the red horizontal line, represent putative loci under selection. Outlier SNPs detected by both F_{ST} and PCAdapt that received functional annotation are highlighted in red, while those without functional annotation are highlighted in green.

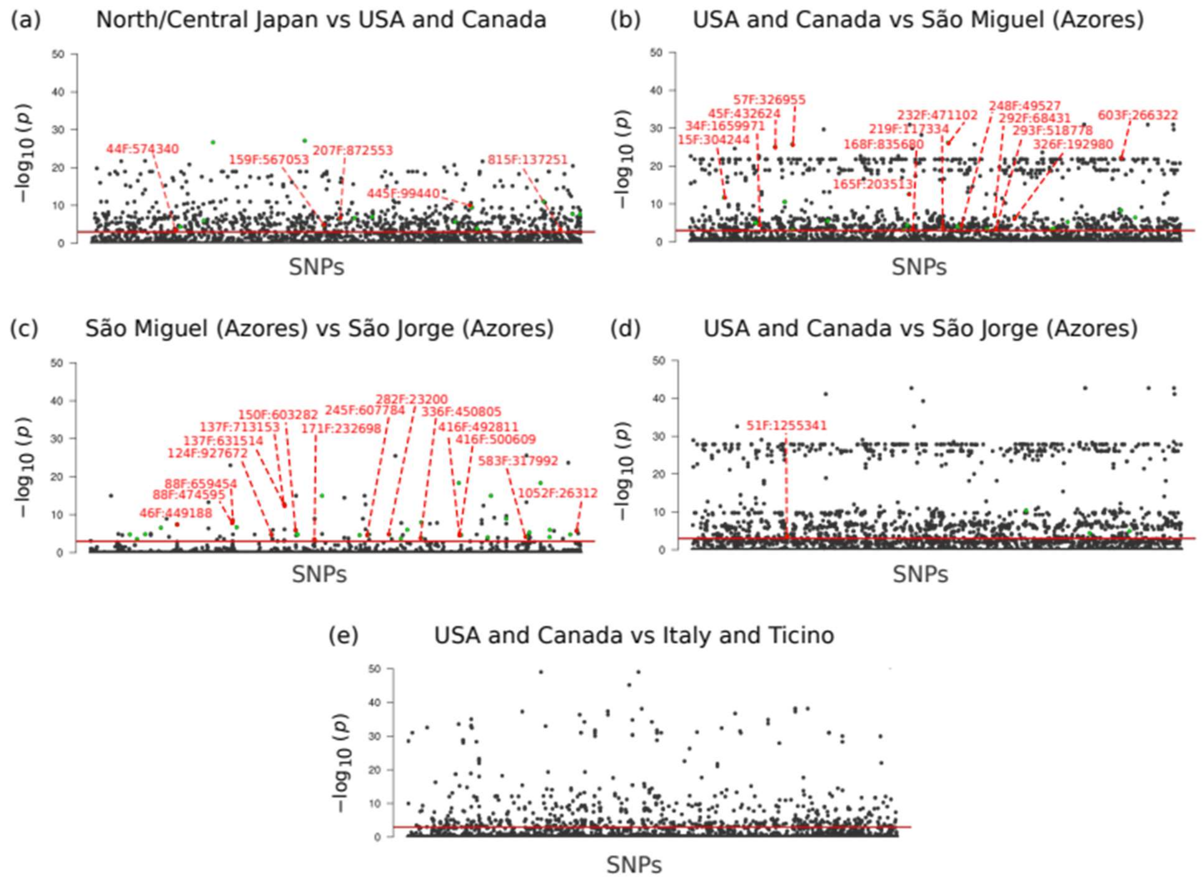

Supplement: Supplementary file 1 — Data S1. [file MEC-34-e70008-s002.pdf]
